# Cyclophilin acts as a ribosome biogenesis factor by chaperoning the ribosomal protein (PlRPS15) in filamentous fungi

**DOI:** 10.1093/nar/gkab1102

**Published:** 2021-11-18

**Authors:** Chenmi Mo, Chong Xie, Gaofeng Wang, Tian Tian, Juan Liu, Chunxiao Zhu, Xueqiong Xiao, Yannong Xiao

**Affiliations:** State Key Laboratory of Agricultural Microbiology, Huazhong Agricultural University, Wuhan 430070, People's Republic of China; Hubei Key Laboratory of Plant Pathology, College of Plant Science and Technology, Huazhong Agricultural University, Wuhan 430070, People's Republic of China; Hubei Key Laboratory of Plant Pathology, College of Plant Science and Technology, Huazhong Agricultural University, Wuhan 430070, People's Republic of China; Hubei Key Laboratory of Plant Pathology, College of Plant Science and Technology, Huazhong Agricultural University, Wuhan 430070, People's Republic of China; Hubei Key Laboratory of Plant Pathology, College of Plant Science and Technology, Huazhong Agricultural University, Wuhan 430070, People's Republic of China; Hubei Key Laboratory of Plant Pathology, College of Plant Science and Technology, Huazhong Agricultural University, Wuhan 430070, People's Republic of China; Hubei Key Laboratory of Plant Pathology, College of Plant Science and Technology, Huazhong Agricultural University, Wuhan 430070, People's Republic of China; State Key Laboratory of Agricultural Microbiology, Huazhong Agricultural University, Wuhan 430070, People's Republic of China; Hubei Key Laboratory of Plant Pathology, College of Plant Science and Technology, Huazhong Agricultural University, Wuhan 430070, People's Republic of China; Hubei Key Laboratory of Plant Pathology, College of Plant Science and Technology, Huazhong Agricultural University, Wuhan 430070, People's Republic of China

## Abstract

The rapid transport of ribosomal proteins (RPs) into the nucleus and their efficient assembly into pre-ribosomal particles are prerequisites for ribosome biogenesis. Proteins that act as dedicated chaperones for RPs to maintain their stability and facilitate their assembly have not been identified in filamentous fungi. PlCYP5 is a nuclear cyclophilin in the nematophagous fungus *Purpureocillium lilacinum*, whose expression is up-regulated during abiotic stress and nematode egg-parasitism. Here, we found that PlCYP5 co-translationally interacted with the unassembled small ribosomal subunit protein, PlRPS15 (uS19). PlRPS15 contained an eukaryote-specific N-terminal extension that mediated the interaction with PlCYP5. PlCYP5 increased the solubility of PlRPS15 independent of its catalytic peptide-prolyl isomerase function and supported the integration of PlRPS15 into pre-ribosomes. Consistently, the phenotypes of the *PlCYP5* loss-of-function mutant were similar to those of the *PlRPS15* knockdown mutant (e.g. growth and ribosome biogenesis defects). PlCYP5 homologs in *Arabidopsis thaliana*, *Homo sapiens*, *Schizosaccharomyces pombe*, *Sclerotinia sclerotiorum*, *Botrytis cinerea* and *Metarhizium anisopliae* were identified. Notably, PlCYP5-PlRPS15 homologs from three filamentous fungi interacted with each other but not those from other species. In summary, our data disclosed a unique dedicated chaperone system for RPs by cyclophilin in filamentous fungi.

## INTRODUCTION

Ribosomes are representative macromolecules that exist in all biological cells. They are essentially responsible for cellular protein synthesis (1). Ribosomes consist of two subunits: large and small. In *Saccharomyces cerevisiae*, the large subunit (60S) contains 25S, 5.8S and 5S rRNA and 46 ribosomal proteins (RPs), while the small subunit (40S) harbors 18S rRNA and 33 RPs (2). Ribosomes mature through a rapid and orderly assembly process to satisfy the protein requirement of biological cells. This process primarily occurs in the nucleus, where the 35S rRNA precursor (pre-rRNA) co-transcriptionally recruits specific RPs and numerous assembly factors to generate the 90S processome (3–5). After progressive cleavages of pre-rRNA, the 90S dissociates into the precursors of the large (pre-60S) and small ribosomal subunits (pre-40S) (3). These two precursors are immediately transported to the cytoplasm, where they associate with additional RPs to form mature ribosome (6–8). Interference with any of these processes results in defective ribosome biogenesis (9).

Efficient nuclear transport and assembly of RPs are crucial for ribosome biogenesis. However, little is known about the mechanisms that mediate the transport of RPs from cytoplasm to nucleus. Newly synthesized RPs tend to aggregate due to their ever-present basic regions and unfolded extensions prone to nonspecific interactions (10). Accordingly, cells employ a general chaperone system, such as the nascent polypeptide-associated complex (NAC), stress 70-B/ribosome-associated complex (SSB/RAC), and importins to protect these aggregate-prone RPs (11–14). Moreover, different chaperones in *S*. *cerevisiae* associate with and solubilize specific RPs, which facilitates nuclear import of RPs and their integration into pre-ribosome, known as dedicated chaperones (15). Five chaperones interact with the RPs of 60S, including Rrb1, Acl4, Sqt1, Syo1 and Bcp1 (15–19), and three chaperones interact with the RPs of 40S, including Yar1, Tsr2 and Tsr4 (20–23). Remarkably, the function as a dedicated RP chaperone appears to be conserved among homologs across species. For instance, a chaperone, the arginine methyltransferase 3 in *Arabidopsis thaliana* and *Schizosaccharomyces pombe* interacts with RPS2 and regulates ribosome biogenesis (24,25). A human chaperone, PDCD2 as well as its yeast homolog Tsr4, co-translationally interacts with RPS2 to facilitate its assembly (23,26). However, it is unclear whether this dedicated chaperone system is conserved in filamentous fungi.

Cyclophilins (CYPs) are ubiquitous proteins and belong to the peptidyl-prolyl cis-trans isomerases (PPIases) that exhibit catalytic activity in protein folding (27). With this feature, CYPs are involved in many biological processes across multiple species, such as cell morphogenesis, transcriptional regulation, abiotic stress resistance, and virulence (28–31). In addition, CYPs possess PPIase-independent chaperone-like activity, which prevents the aggregation of multiple proteins through direct binding (32–34). Recently, the biological function of CYPs as chaperones has been emphasized. The plant CYP40 acts as a co-chaperone of Hsp90 to promote microRNA activities by facilitating the binding of small ligands to Ago1 (35). The CYP40 homolog in *Drosophila melanogaster* has also been identified as the Hsp90 co-chaperone, which is essential for spermatogenesis and modulation of Ago2-RISC formation (36). Nevertheless, other possible chaperone functions of CYPs remain to be explored.

*Purpureocillium lilacinum* is a filamentous fungus of the phylum Ascomycota and is widely used to control plant-parasitic nematodes due to its ability to parasitize nematode eggs and the nematocidal activity of fungal metabolites (37–40). However, the molecular mechanism underlying its parasitism remains unclear, which undermines the field application of this fungal biocontrol agent. In our previous study, we identified the CYP family of *P. lilacinum* and found the gene expression of *PlCYP5*, an RNA recognition motif (RRM)-containing *CYP* gene, was increased upon infection of nematode eggs (41). In addition, it was also increased upon exposure to abiotic stressors (41), implying versatile roles of *PlCYP5*. Surprisingly, the model fungus *S*. *cerevisiae* lacks the homolog of PlCYP5. Its homologs in *A. thaliana* and *S. pombe* regulate transcription by interacting with the C-terminal domain of RNA polymerase II (42,43). However, there was no such interaction in *P*. *lilacinum*. Here, we aimed to identify a novel function of the RRM-containing CYP and determine its effect on cellular ribosome biogenesis.

## MATERIALS AND METHODS

### Strains and growth condition

The *P. lilacinum* wildtype strain 36-1 was isolated from the egg surface of *M. incognita* and was cultured on PDA at 28°C for reproduction and on CZA at 28°C for biological phenotype determination. Gene mutation strains were cultured on potato dextrose agar (PDA) supplemented with 1.2 mg/ml G418 sulfate and gene complementary strain was cultured on PDA supplemented with 2 mg/ml glufosinate ammonium. *Escherichia coli* strain was cultured in Luria-Bertani broth (LB) medium at 37°C.

### Sequence analysis

The conserved protein domain of PlCYP5 was identified using the PROSITE (https://prosite.expasy.org/). Sequence multiple alignment was performed using the MUSCLE program of MEGA 7.0 with default parameters, and the alignment result was applied to generate a phylogenetic tree with 1000 bootstraps. For protein structure prediction, the amino acid sequences of proteins were submitted to I-TASSER server (https://zhanglab.ccmb.med.umich.edu/I-TASSER/), and the most reliable result was utilized.

### Fungal transformation

Transformation of *P. lilacinum* was performed based on the polyethylene glycol (PEG)-mediated protoplast transformation described previously with some modifications (44,45). Briefly, to produce protoplasts, 50 μl of a 1 × 10^5^ conidia/mL suspension of wildtype strains was inoculated into 100 ml tryptone-glucose medium (10 g/l tryptone, 10 g/l glucose) and shaken at 28°C. At 2 dpi (day post-inoculation), the mycelium was collected by filtering through three layers of lens paper and washing with 0.7 M NaCl to remove conidia and medium. The mycelium was then digested with 10 mg/ml lysing enzyme (Sigma, USA) and 1 mg/ml snailase (Solarbio, China) mixed solution at 120 rpm at 30°C for 4 h. The protoplasts were harvested by filtering through three layers of lens paper and washing with 0.7 M NaCl. After centrifugation at 5000 rpm at 4°C for 6 min, the protoplasts were suspended in STC solution (1.2 M sorbitol, 10 mM Tris–HCl (pH 7.5), 50 mM CaCl_2_) and adjusted to the concentration of 1 × 10^8^ protoplasts/ml.

For the transformation, 100 μl of protoplasts were mixed with 2–5 μg of DNA fragments, and TEC solution (10 mM Tris–HCl (pH 7.5), 1 mM ethylene diamine tetraacetic acid (EDTA), 50 mM CaCl_2_) was added up to 160 μl. The sample was gently mixed and incubated with ice for 20 min. 160 μl of 60% PEG 3350 dissolved in 0.12 M 4-morpholinepropanesulfonic acid was added dropwise and incubated at 28°C for 30 min. After incubation, 1 ml of STC was added and mixed gently. The mixture was centrifuged at 5000 rpm at 4°C for 6 min to recover the protoplasts, which were then suspended in 250 μl STC. Each 50 μl of protoplasts was spread on a PDA plate supplemented with 10 g/l molasses, 0.6 M/l sucrose, 0.3 g/l yeast extract, 0.3 g/l tryptone and 0.3 g/l casein peptone, and incubated at 28°C. After 24 h, the plates were overlaid with T-top medium containing 1.2 mg/ml G418 sulphate, and the transformants were selected 2–7 days later.

### Subcellular localization

All primers used in this study were listed in Supplementary Table S1. The promoter of *gpdP* gene, which was the homologous gene of *Aspergillus nidulans gpdA* in *P*. *lilacinum*, was amplified with the primer pairs PgpdP-F/PgpdP-R. The primer pairs, eGFP-F/eGFP-R and PlCYP-F/PlCYP5-R, were used to clone the enhanced green fluorescent protein (*eGFP*) and *PlCYP5* gene, respectively. Overlap extension PCR was conducted to fuse these three fragments with primer pairs PgpdP-F/PlCYP5-R, generating the PgpdP::*eGFP*::*PlCYP5* expression cassette. To observe the localization of truncated PlCYP5, PlCYP5-F/PlCYP5_1-331_-R and PlCYP5_332-478_-F/PlCYP5-R were used to amplify *PlCYP5_1-331_* and *PlCYP5_332-478_* fragments, respectively and fused using the same strategy. PgpdP::*eGFP* expression cassette was used as control. Transformants were screened on medium with 1.2 mg/ml G418 and further identified via PCR using the primer pairs, PgpdPJ-F/PlCYP5J-R. The blastospores and hyphae were visualized under a confocal laser scanning microscope.

### Gene knockout and complementation

Approximately 1.3 kb of upstream and downstream fragments of the *PlCYP5* gene was cloned with the primer pairs, PlCYP5L-F/PlCYP5L-R and PlCYP5R-F/PlCYP5R-R, respectively, which were named as *C5L* and *C5R*. The G418 resistance gene *NPTII* was split into two fragments, *NP* and *PT*, with 1.0 kb repetitive region and cloned with primer pairs, NP-F/NP-R and PT-F/PT-R. Overlap extension PCR was then conducted to generate the *C5LNP* and *PTC5R* fragments with primer pairs C5L-F/NP-R and PT-F/C5R-R, respectively. The *C5LNP* and *PTC5R* were co-transformed into wildtype protoplasts. Transformants were screened with 1.2 mg/ml G418. Gene replacement required four primer pairs for verification. C5LJ-F/NPJ-R and PTJ-F/C5RJ-R verified the upstream and downstream regions, respectively. NP-F/PT-R verified the *NPTII* gene, and PlCYP5J-F/PlCYP5J-R verified the target gene. The same strategy was used for the knockout of the *PlRPS15* gene.

### Biological phenotype experiments

To assess differences in growth, aliquots of 1.5 μl 1 × 10^5^ conidia/mL suspension of each strain were spotted on the center of CZA and PDA plates. All spotted plates were incubated at 28°C for 14 days, followed by measuring the diameter of each colony.

To assess the response of each strain to abiotic stress, the same spotted method was conducted. CZA plates were supplemented with the following chemicals: 1 M NaCl, 1 M KCl, 1.2 M sorbitol, 0.1% SDS, 0.15 mg/ml Congo red or 5 mM H_2_O_2_. After incubation at 28°C for 14 days, the diameter of each colony was measured.

The conidia production capacity of each strain was determined by spreading 100 μl of a 1 × 10^7^ conidia/ml suspension per CZA plate. After 14 days of dark culture at 28°C, three plugs (6 mm diameter) were bored from each plate using a puncher, and the conidia of the three plugs were released into 1 ml of 0.02% Tween 80 through vibration. The conidial concentration was quantified using a hemocytometer and converted to the number of conidia per cm^2^ plate culture. The blastospore production capacity of each strain was determined by inoculating two fresh plugs into 150 ml PDB. After shaking at 180 rpm for 5 days, the culture was filtered through three layers of lens paper. The blastospore concentration was quantified using a hemocytometer and converted to the number of blastospores per ml. All experiments were conducted in triplicates.

### RNA-seq and real-time quantitative PCR (RT-qPCR) analysis

For RNA-seq, 50 μl of 1 × 10^5^ conidia/ml suspensions of wildtype and Δ*PlCYP5* were inoculated into 100 ml PDB medium respectively, followed by shaken at 28°C to collect the mycelia at vegetative growth stage. Because Δ*PlCYP5* grew more slowly than wildtype, the mycelia of wildtype were collected at 2 dpi, while Δ*PlCYP5* were collected at 4 dpi. The mycelia were filtered through three layers of lens paper and washed with water to remove the medium. After removing excess water, the total RNA of the samples was extracted with RNAiso Plus kit (Takara, China). After RNA quality inspection, sequencing was conducted on an Illumina MiSeq sequencing system following the manufacturer's instructions. *P. lilacinum* 36–1 genome in the database (ftp://ftp.ncbi.nlm.nih.gov/genomes/all/GCA/003/144/605/GCA_003144605.1_ASM314460v1) was used as a reference for mapping the reads using HISAT2 v2.0.1 to obtain reads count (46). The counts were normalized by HTSeq v0.6.1, and differentially expressed genes were identified using the edgeR package with the false discovery rate (FDR) < 0.05 and |log_2_(FoldChange)| ≥ 1. The Blast2GO program was used to get Gene ontology (GO) annotation.

The total RNA used for RNA-Seq was also employed to analyze the expression levels of the target genes by RT-qPCR. The RNA samples were treated with the DNA-free™ DNA Removal Kit (Invitrogen™, USA) to remove DNA, and then the RNA was used to generate the first strand of cDNA with the RevertAid First Strand cDNA Synthesis Kit (Thermo Scientific, USA). Gene expression abundance was analyzed using the Bio-Rad CFX96 Real-Time System and SsoFastTMEvaGreen Supermix (Bio-Rad, Hercules, CA, USA). The fold change of gene expression was calculated in comparison with the control by the 2^−ΔΔCt^ method.

### Protein interaction assays

For the Y2H assay, plasmid pGBKT7 (BD) expressing bait protein and plasmid pGADT7 (AD) expressing prey protein were co-transformed into the yeast strain Y2H GOLD (Clonetech, China). Transformants were screened by SD/-Trp-Leu and validated by PCR using general primers of BD and AD. The interactions were assessed by spotting transformants in 10-fold dilution onto SD/-Trp-Leu supplemented with X-α-galactosidase (X-α-gal) and Aureobasidin A (AbA) and SD/-Trp-Leu-His-Ade supplemented with X-α-gal and AbA, followed by incubation for 3 days at 30°C.

To analyze the interactions between PlCYP5 and PlRPS15 *in vivo*, Co-immunoprecipitation (Co-IP) was performed as follows. GFP-PlCYP5 and myc-PlRPS15 were co-expressed in *P. lilacinum* wildtype. The fresh mycelium was collected and ground into powder in liquid nitrogen. Total protein was isolated by adding RIPA lysis buffer (Beyotime, China) supplemented with 1 mM phenylmethanesulfonyl fluoride (PMSF) and 1% proteinase inhibitor cocktail (Sigma, USA) and then centrifuged at 13 000 rpm at 4°C for 30 min to remove cell debris. The protein extract was subjected to IP assay using anti-GFP affinity sepharose (Dia-an, China). After incubating at 4°C for 8 h, the sepharose was washed three times with RIPA lysis buffer. The bound proteins were eluted by boiling for 5 min in protein loading buffer and subjected to immunoblot with c-myc antibody (Proteintech, USA). GFP co-expressed with myc-PlRPS15 in wildtype was used as control.

To validate the direct interactions between PlCYP5 and PlRPS15, or between truncated PlCYP5 and PlRPS15, the open reading frame of *PlCYP5* or its truncations was cloned into plasmid pGEX-6P-1 containing a GST tag. Then the plasmid was transformed into *E. coli* Rosetta (DE3). Cells were grown in 50 ml of LB medium and protein expression was induced at an OD_600_ of 0.6–0.8 by the addition of isopropyl-beta-d-thiogalactoside to a final concentration of 0.2 mM. After cultured at 16°C overnight, cells were suspended in 5 ml PBS supplemented with 1 mM phenylmethylsulfonyl fluoride (PMSF) and lysed ultrasonically. Recombinant GST-PlCYP5 (or truncated PlCYP5) was immobilized on Glutathione Sepharose (GE healthcare, USA), and incubated with *E. coli* lysates containing 6 × *His*-PlRPS15 at 4°C for 2 h. After washed three times with PBS, the bound proteins were eluted with elution buffer (10 mM reduced glutathione, 50 mM Tris–HCl pH 8.0). The eluted proteins were separated by SDS-PAGE and subjected to western blotting using an antibody against 6 × *His* (Proteintech, USA). GST protein incubated with *E. coli* lysates containing 6 × *His*-PlRPS15 was used as a control.

### Polysome profile analysis

Polysome profile analysis of *P. lilacinum* was carried out referring to the method used in the yeast (47). Briefly, 100 μl of a 1 × 10^5^ conidia/ml suspension of each strain was inoculated into 500 ml PDB and cultured to the vegetative growth stage. Then, cycloheximide (CHX) was added into the culture to a final concentration of 500 μg/ml, and the mixture was continuously incubated for another 30 min. Mycelium was collected by filtering through three layers of lens paper and washed with CH buffer (10 mM Tris–HCl, pH 7.5, 100 mM NaCl, 30 mM MgCl_2_, 6 mM β-mercaptoethanol, and 500 μg/ml CHX). Cell extracts were prepared by grinding mycelium with liquid nitrogen and dissolved in CH buffer at 4°C. Then, 6 *A*_260_ units of the cell extracts were loaded onto linear 7–47% sucrose gradients prepared by CH buffer. After 2.5 h of centrifugation at 35000 rpm in a P40ST rotor (Hitachi), gradient fractions were collected and monitored at 254 nm.

### Nascent synthesized protein analysis

Nascent protein synthesis was measured by Click-iT^®^ protein reaction system (Thermo Fisher, USA) according to the manufacturer's protocol. Briefly, the blastospores of each strain were produced as described above to a concentration of 1 × 10^7^ blastospores/ml and recovered in methionine-free Czapek-Dox liquid medium at 28°C for 1 h to deplete the methionine reserves. To provide a reference, a final concentration of 500 ug/ml CHX was added to the medium before recovering. Blastospores were then collected by centrifuge and subjected to the Click reaction. The nascent protein level was assessed by determining signal intensity using a fluorescence microplate reader. The experiment was repeated three times.

### Northern blot

Total RNA of *P*. *lilacinum* was prepared by the standard method using TRIzol reagent (Takara, China). The appropriate amount of total RNAs was separated on 1.2% formaldehyde denaturing agarose gel. RNAs were then transferred onto nylon membranes and then cross-linked to the membrane by UV. Hybridization was performed overnight at 50°C using the following labelled DNA probes (GE Healthcare): 18S (5′-CTACTACATCCAAGGAAGGCAGCAGGCGCGCAAATTACCCAATCCCGACAC-3′), 28S (5′-GGAGTCGTCTTCGTATGCGAGTGTTCGGGTGTAAAACCCCTACGCGTAAT-3′), 5′-ETS (5′-CCACCAGTAACTTGGAAAATCTCTCCGGCGCTGAAACACGCGCCGGTAGGCCA-3′) and ITS1 (5′-CGAGTTATACAACTCCCAAACCCACTGTGA ACCTTACCTCAGTTGCCTCGG-3′). The membranes were washed three times for 10 min at 50°C in wash buffer (GE Healthcare), and signals were detected using ChemiDoc Touch Imaging System (Bio-rad, USA).

### PPIase assay

PPIase assay was performed as previously described using the tetrapeptide substrate Suc-AAPF-pNA (*N*-succinyl-Ala-Leu-Pro-Phe p-nitroanilide, Sigma) (33), with the following modifications: The activity assay mixture consists of 215 μl 50 mM HEPES buffer (containing 100 mM NaCl), 25 μl 10 mg/ml α-chymotrypsin and 5 μl appropriate PPIase protein. The assay was initiated by adding 5 μl of Suc-AAPF-pNA solution to a volume of 250 μl. Absorbance at 390 nm was recorded every 3 s for a duration of 5 min. The experiment was repeated four times.

### Protein aggregation assay

To test the function of PlCYP5 against PlRPS15 aggregation *in vivo*, the sequences of *PlCYP5* gene without the CR (*PlCYP5_1-993_*) and *PlRPS15* gene were cloned into plasmid pETDuet-1 together and then co-expressed in the *E. coli* strain Rosetta (DE3). After induction with 0.2 mM IPTG in 20 mL LB as described above, cells were suspended in 2 mL PBS supplemented with 1 mM PMSF and lysed ultrasonically for 5 min. To separate supernatant and pellet, the cell lysate was centrifuged at 13 000 rpm at 4°C for 10 min, and the pellet was resuspended in 2 mL PBS. All samples were separated by SDS-PAGE and analyzed by Coomassie Blue staining. *E. coli* strains expressing *PlCYP5_1-993_* and *PlRPS15* alone were used as control.

### Statistical analysis

The statistical significance of differences was examined using the two-way ANOVA analysis, followed by Bonferroni's post-test. Data were presented as the mean ± standard deviation (SD) and the corresponding *p-value* was indicated in the figure legends.

## RESULTS

### PlCYP5 is a nuclear RRM-containing CYP

**Figure 1.**
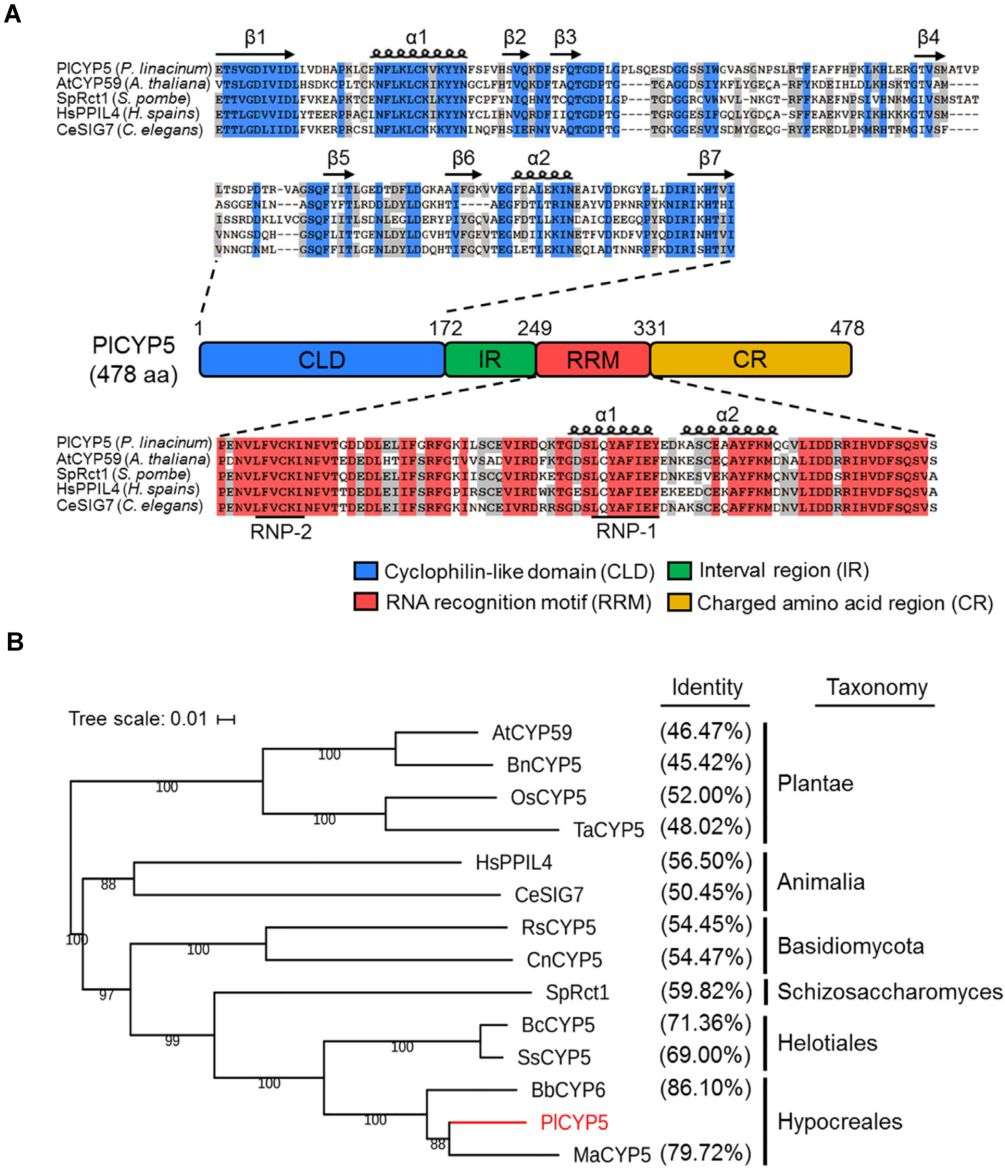
PlCYP5 is a conserved RRM-containing CYP. (**A**) Multiple sequence alignment of the CLD and RRM domains of the RRM-containing CYPs in different species. Amino acids with different colored background indicate different conservation. The secondary structures were displayed above the sequences. β indicates the β fold, and α indicates the α helix. (**B**) Phylogenetic analysis of PlCYP5 with its homologs in different species. Sequence identity between PlCYP5 and its homologs was obtained by Blastp comparison and showed behind each label. At: *Arabidopsis thaliana*, Bn: *Brassica napus*, Os: *Oryza sativa*, Ta: *Triticum aestivum*, Hs: *Homo sapiens*, Ce: *Caenorhabditis elegans*, Rs: *Rhizoctonia solani*, Cn: *Crytococcus neoformans*, Sp: *Schizosaccharomyces pombe*, Bc: *Botrytis cinerea*, Ss: *Sclerotinia sclerotiorum*, Bb: *Beauveria bassiana*, Pl: *Purpureocillium lilacinum*, Ma: *Metarhizium anisopliae*.

Two characteristic sequences in PlCYP5 were identified by functional domain analysis; 1–172 amino acids (aa) formed the cyclophilin-like domain (CLD) and 249–331 aa comprised the RNA recognition motif (RRM) (Figure 1A). Hence, we defined the 173–248 aa with no functional annotation as the interval region (IR), and the charged amino acid-enriched region after RRM as the charged region (CR) (Figure 1A). The CLD and RRM of PlCYP5 are highly conserved among its homologous proteins, while the IR and CR are poorly conserved (Figure 1A, Supplementary Figure S1). Phylogenetic analysis showed that PlCYP5 was clustered together with its homologs from the Hypocreales fungi, suggesting that they have a similar function (Figure 1B).

**Figure 2.**
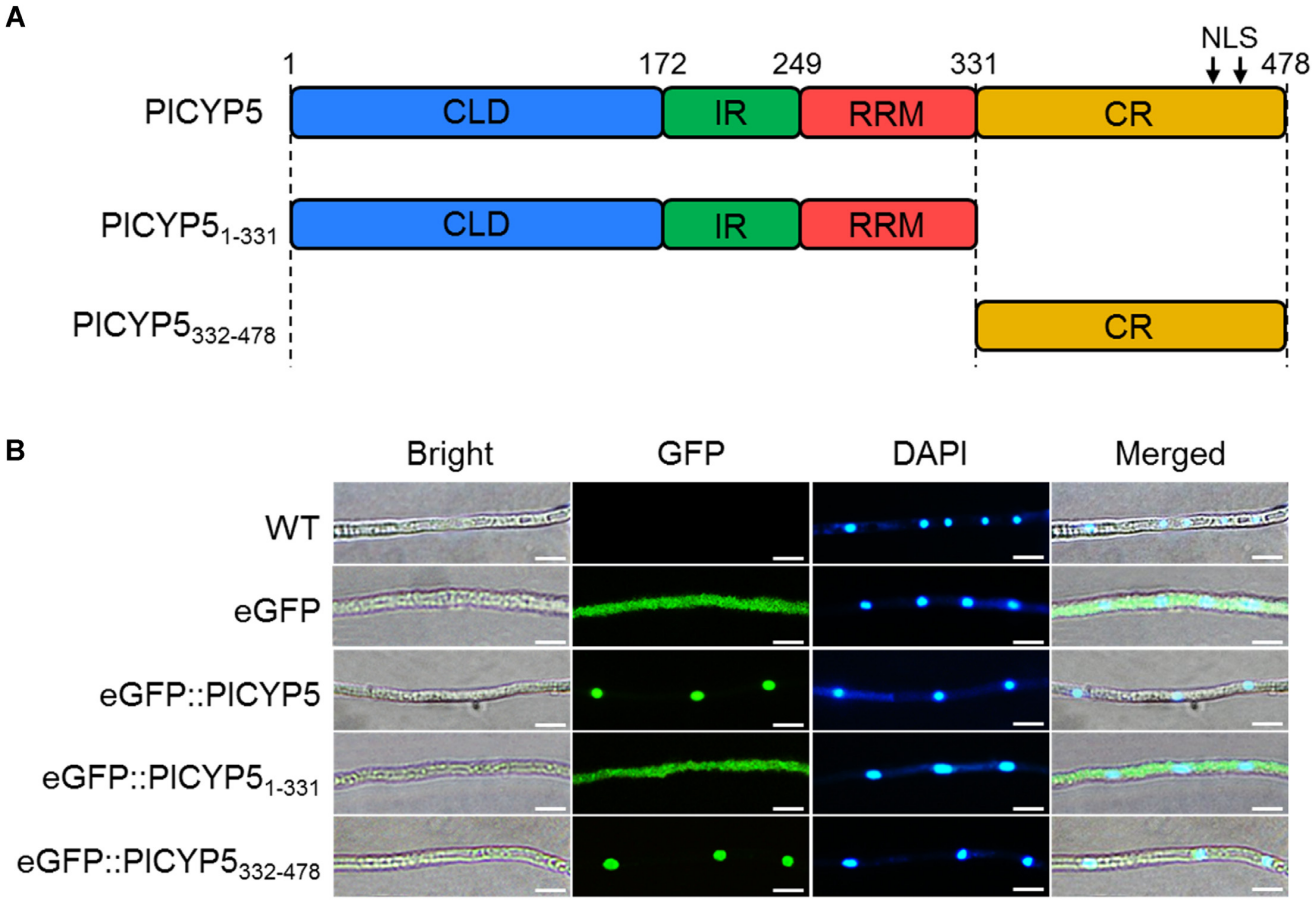
PlCYP5 is located in the nucleus. (**A**) Schematic diagram of PlCYP5 truncation. (**B**) Subcellular localization of PlCYP5 and its truncation in the hyphae of *P*. *lilacinum*. Fresh hyphae of wildtype and transformants were collected and visualized under a confocal microscope. 4’,6-diamidino-2-phenylindole (DAPI) was used to stain the nucleus. Scale bar = 10 μm.

In the previous study, we demonstrated that PlCYP5 localized in the nucleus when transiently expressed as enhanced green fluorescent protein (eGFP)::PlCYP5 fusion protein in *Nicotiana benthamiana* (41). The nuclear localization of PlCYP5 was further confirmed by expressing the full length of PlCYP5 fused with eGFP in the hyphae of *P. lilacinum* (Figure 2B). PlCYP5 harbored two nuclear localization signals (NLSs) at the C-terminal (Figure 2A). Deletion of CR containing the NLSs (PlCYP5_1-331_) prevented PlCYP5 from entering the nucleus, while CR (PlCYP5_332-478_) alone localized in the nucleus, pointing to the importance of the NLSs for PlCYP5 nuclear localization (Figure 2B).

### PlCYP5 is involved in the growth, development, and virulence of *P. lilacinum*

**Figure 3.**
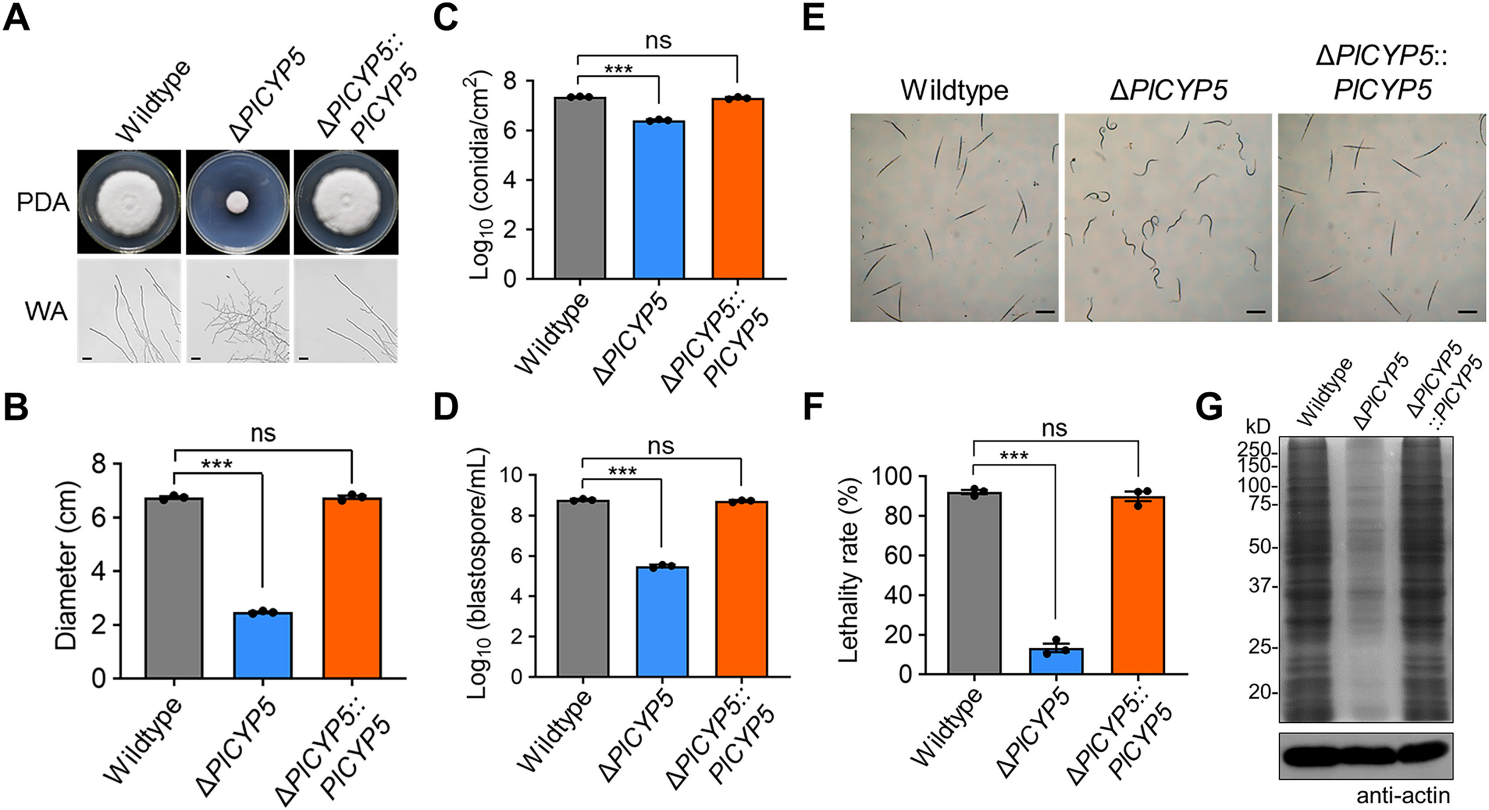
Δ*PlCYP5* shows defects in growth, development, and virulence. (**A**) Images of growth morphology of wildtype, Δ*PlCYP5*, and Δ*PlCYP5*::*PlCYP5* strains. Colony growth of each strain was observed after 14 days of culture on potato dextrose agar (PDA) plates. Hyphae tips were observed after 4 days of culture on water agar (WA) plates. Scale bar = 50 μm. (**B**) Colony diameters of wildtype, Δ*PlCYP5* and Δ*PlCYP5*::*PlCYP5* strains after 14 days of culture on PDA plates. (**C**, **D**) Conidia (C) and blastospores (D) yields of the wildtype, Δ*PlCYP5*, and Δ*PlCYP5*::*PlCYP5* strains. (**E**) Images of nematode survival in the fermentation of wildtype, Δ*PlCYP5* and Δ*PlCYP5*::*PlCYP5* strains. Fermentations of strains at the same biomass were collected by centrifuge, followed by filtering through 0.22 μm membrane. About 100 second juveniles of *Meloidogyne incognita* were inoculated into the fermentation of each strain and incubated for 3 days. Scale bar = 200 μm. (**F**) The lethality rate of fermentations of wildtype, Δ*PlCYP5* and Δ*PlCYP5*::*PlCYP5* strains to nematodes. (**G**) SDS-PAGE separation of total proteins in the fermentation of wildtype, Δ*PlCYP5* and Δ*PlCYP5*::*PlCYP5* strains. All the cultured mycelia of each strain were collected to extract proteins, and then one-third of the protein extracts were used to hybridize with actin antibody. The error bars indicate the SD of three replicates. *** denotes *P* < 0.001.

To explore the function of *PlCYP5*, the gene deletion (Δ*PlCYP5*) and complementary (Δ*PlCYP5::PlCYP5*) strains were obtained through homologous recombination and verified via Southern blotting (Supplementary Figure S2). The Δ*PlCYP5* strain showed a significantly attenuated growth on the potato dextrose agar (PDA) and Czapek-Dox agar (CZA) plates (Figure 3A and B). When cultured on the water agar plates, Δ*PlCYP5* displayed thinner hyphal tips and hyperbranching compared with the wildtype and Δ*PlCYP5*::*PlCYP5* (Figure 3A). The conidia and blastospore production of Δ*PlCYP5* was significantly decreased compared with those of the wildtype and Δ*PlCYP5*::*PlCYP5* (Figure 3C and D). Moreover, the Δ*PlCYP5* strain had an increased sensitivity to abiotic stresses (Supplementary Figure S3).

As a nematode bio-control fungus, the nematocidal activity of wildtype and mutant strains was also investigated. To exclude the effect of growth differences between strains on fermentation, we determined the time point for each strain reaching the same biomass, and then collected their fermentations with equal biomass. After incubation of the second juveniles of the nematode *Meloidogyne incognita* with fungal fermentation for 72 h, more than 90% of the second juveniles with the fermentation of wildtype and Δ*PlCYP5*::*PlCYP5* were dead, while Δ*PlCYP5* fermentation showed significantly weak activity (Figure 3E and F). We further compared the total protein content in the fermentation of those strains and found that the amount of proteins in Δ*PlCYP5* fermentation was significantly less than that of the wildtype and Δ*PlCYP5*::*PlCYP5* (Figure 3G).

To determine the functional domain of PlCYP5, we divided PlCYP5 into four segments, including CLD, IR, RRM and CR, based on the domain analysis (Figure 1A). To ensure that each fragment localizes in the nucleus as the full length PlCYP5, the CLD, IR and RRM were fused with CR containing the NLSs (Supplementary Figure S4A). Each fused fragment was expressed in Δ*PlCYP5* strain. Intriguingly, the growth defect caused by *PlCYP5* gene deletion could be rescued by none of the fused fragments (Supplementary Figure S4B), suggesting that each part of *PlCYP5* was indispensable for its function.

### Differentially expressed genes in Δ*PlCYP5* were enriched in ribosome biogenesis

**Figure 4.**
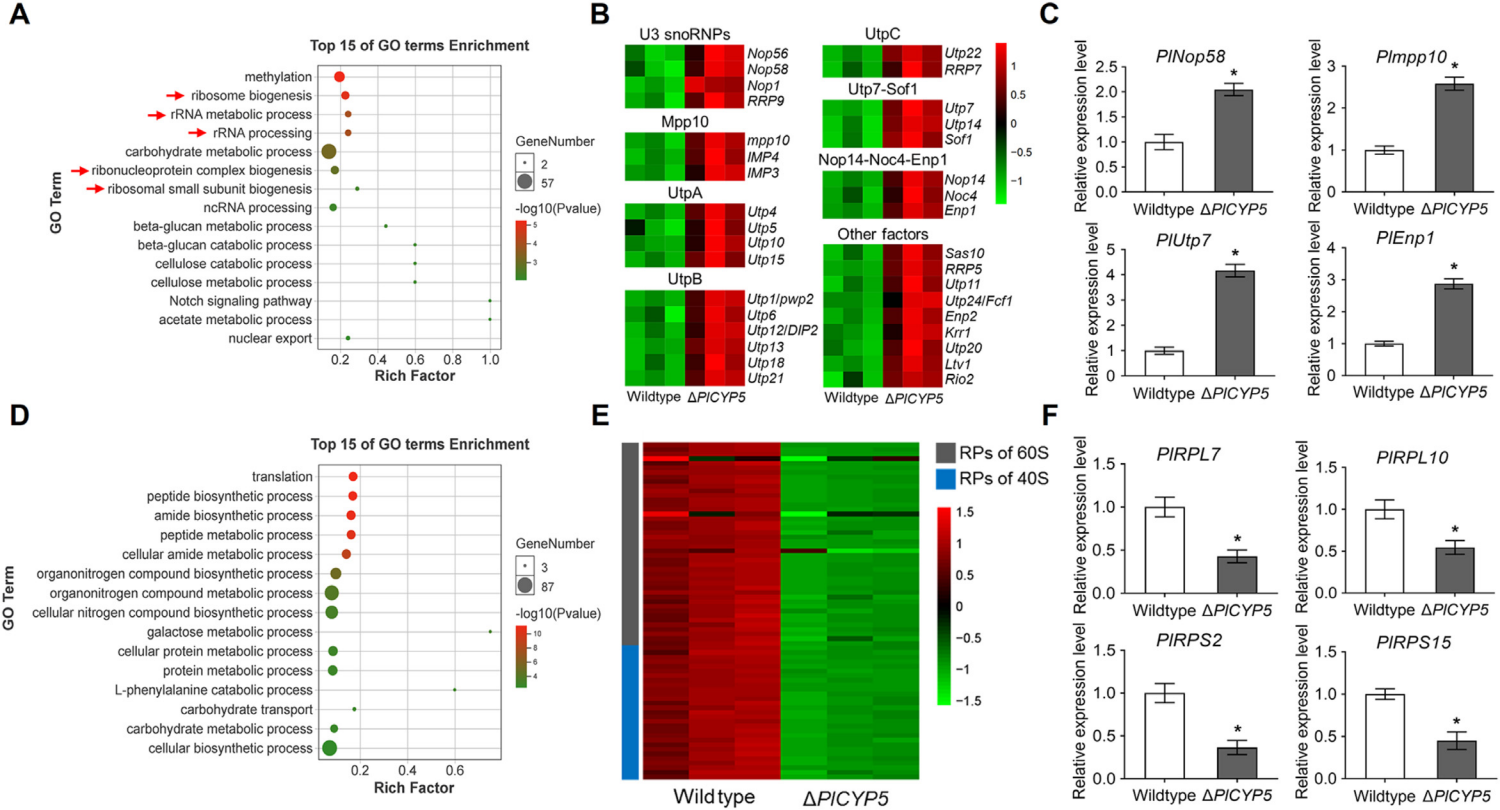
Genes related to ribosome biogenesis were up-regulated in Δ*PlCYP5* strain. (**A**) GO enrichment in the biological process of up-regulated genes in Δ*PlCYP5*. Red arrows indicate the biological processes related to ribosome biogenesis. (**B**) Gene expression of ribosome biogenesis factors in transcriptome data. (**C**) RT-qPCR verification of the selected ribosome biogenesis factor genes. (**D**) GO enrichment in the biological process of down-regulated genes in Δ*PlCYP5*. (**E**) Gene expression of ribosomal proteins in transcriptome data. (**F**) RT-qPCR verification of selected ribosomal protein genes. The error bars indicate the SD of three replicates. * denotes *P* < 0.05.

For an in-depth understanding of the biological function of *PlCYP5*, transcriptome analysis of wildtype and Δ*PlCYP5* strains was performed. A total of 2117 differentially expressed genes were identified in Δ*PlCYP5* compared with the wildtype. Of these, 1404 genes were upregulated, while 713 genes were downregulated in Δ*PlCYP5* (Supplementary Figure S5). Gene ontology (GO) enrichment showed that the upregulated genes had functions associated with ribosome biogenesis, including rRNA metabolic process, rRNA processing, ribonucleoprotein complex biogenesis, and ribosomal small subunit biogenesis (Figure 4A). Consistent with the GO enrichment analysis, genes encoding ribosomal assembly factors, including *PlNop58*, *Plmpp10*, *PlUtp7* and *PlEnp1*, were upregulated in Δ*PlCYP5* (Figure 4B). These were verified by RT-qPCR (Figure 4C). The carbohydrate metabolic process was also enriched and had the largest number of genes (Figure 4A), possibly because ribosome biogenesis was a major energy consuming process (48). The downregulated genes were enriched in translation and various metabolic and biosynthetic processes (Figure 4D). Interestingly, although the genes of ribosomal assembly were upregulated, the RP genes of both 60S and 40S were significantly downregulated (Figure 4E and F), which implied that the ribosomes in mutant cells were impaired. Therefore, we presumed that PlCYP5 plays a role in ribosome biogenesis.

### PlCYP5 physically interacted with unassembled PlRPS15

**Figure 5.**
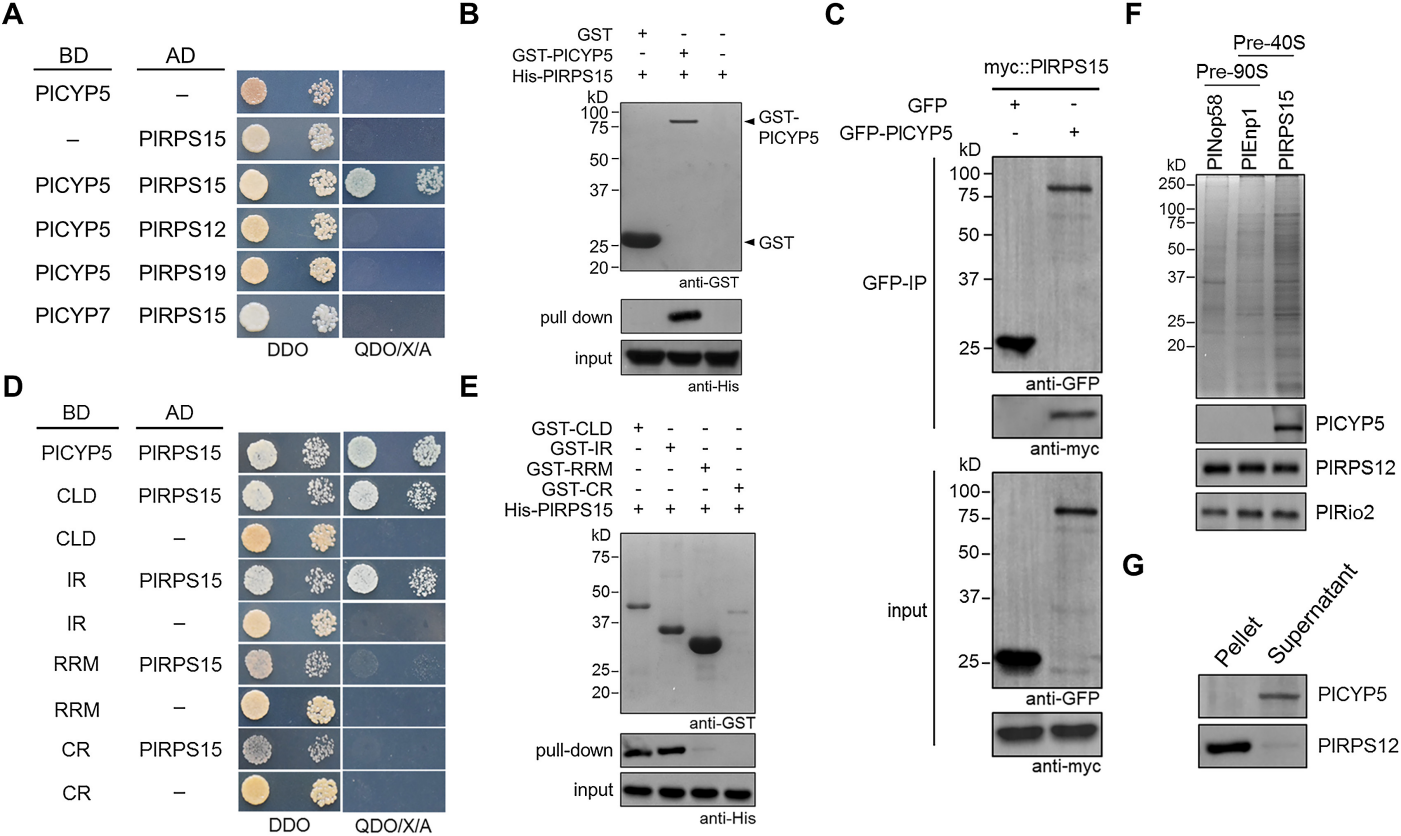
PlCYP5 directly binds to unassembled PlRPS15. (**A**) Y2H interaction between PlCYP5 and PlRPS15. The specific interaction of PlCYP5 and PlRPS15 was demonstrated by negative interactions of PlCYP5 with other RPs (PlRPS12 and PlRPS19) and PlCYP7 with PlRPS15. DDO: SD/-Trp-Leu. QDO/X/A: SD/-Trp-Leu-His-Ade supplemented with X-α-gal and aureobasidin A (AbA). (**B**) GST pull-down assay between PlCYP5 and PlRPS15. The GST tagged PlCYP5 was expressed in *E*. *coli* and purified via Glutathione Sepharose. Proteins were separated by SDS-PAGE, followed by western blot analysis using GST or 6 × *His* antibodies. The bands highlighted by black arrowheads correspond to the GST and GST-PlCYP5 proteins used as baits. (**C**) Co-immunoprecipitation (Co-IP) analysis of PlCYP5 and PlRPS15. The eGFP::PlCYP5 and myc::PlRPS15 were co-expressed in *P*. *lilacinum*. The protein extract was purified via anti-GFP affinity sepharose and separated by SDS-PAGE, following by western blot analysis using GFP and c-myc antibodies. (**D**) Y2H interaction between truncated PlCYP5 and PlRPS15. PlCYP5 was divided into four truncations, including the CLD, IR, RRM, and RS based on protein domain analysis. (**E**) GST pull-down assay between truncated PlCYP5 and PlRPS15. (**F**) PlCYP5 is not associated with pre-ribosome. 3 × *flag*-tagged PlNop58 and PlEnp1 were expressed and purified via anti-*flag* affinity sepharose, respectively. Proteins were separated by SDS-PAGE and performed coomassie blue staining or analyzed by western blot to detect the presence of PlCYP5, PlRPS12 and PlRio2. (**G**) PlCYP5 does not bind to ribosome. Non-ribosomal and ribosome-bound proteins were separated by ultracentrifugal sedimentation. Pellet and supernatant were separated by SDS-PAGE, and analyzed by western blot analysis to detect the presence of PlCYP5 and PlRPS12.

To explore the molecular mechanism in which PlCYP5 plays a role, a pull-down assay coupled with LC/MS was applied to identify PlCYP5-interacting proteins. A total of 79 proteins were identified, of which 54 were predicted to be localized in the nucleus or shuttled between the cytoplasm and the nucleus (Supplementary Table S2). GO enrichment analysis of these interacting candidates disclosed the possible functions of PlCYP5 in protein binding, nucleic acid binding, and transcription regulation (Supplementary Figure S6). Among these candidates, several proteins were RPs of the small subunit, including RPS12 (eS12), RPS15 (uS19) and RPS19 (eS19) (Supplementary Table S2). As the transcriptome analysis suggests the role of PlCYP5 in ribosome biogenesis, we further investigated its interaction with these RPs. In yeast two-hybrid (Y2H) assay, we found that PlCYP5 interacted with PlRPS15 (Figure 5A). The interaction was further validated by *in vitro* pull-down assay and *in vivo* co-immunoprecipitation (Figure 5B and C). In contrast, PlCYP7, another nuclear CYP in *P. lilacinum* (41), did not interact with PlRPS15 (Figure 5A). In addition, PlCYP5 did not interact with PlRPS12 and PlRPS19 (Figure 5A). This indicated that PlCYP5 specifically bound to PlRPS15. To explore which parts of PlCYP5 contribute to the interaction, the CLD, IR, RRM, and CR of PlCYP5 were subjected to interaction assays with PlRPS15. The results of Y2H and pull-down assays showed that the CLD and IR interacted with PlRPS15, while the RRM and CR did not (Figure 5D and E).

Since the newly synthesized RPS15 is incorporated into pre-ribosome during ribosome biogenesis, we investigated whether PlCYP5 binds to PlRPS15 in the pre-ribosome complex or to unassembled PlRPS15. For this purpose, 3 × *flag*-PlNop58, 3 × *flag*-PlEnp1 and 3 × *flag*-PlRPS15 were individually expressed in *P. lilacinum* and purified via *flag* affinity sephorose. We found that all proteins were co-purified with the PlRPS12 and another ribosomal assembly factor, PlRio2 (Figure 5F), indicating that these three proteins from the fungus bound to pre-ribosome as their homologs in *S. cerevisiae*. However, PlCYP5 was co-purified only with PlRPS15 but not with PlNop58 and PlEnp1 (Figure 5F). Moreover, PlCYP5 was present in the non-ribosomal supernatant than in the ribosome precipitation after sucrose ultracentrifugal sedimentation of cell extracts (Figure 5G). These results demonstrated that PlCYP5 was not associated with pre-ribosome but bound to unassembled PlRPS15.

### Disruption of *PlRPS15* phenocopied the *PlCYP5* mutant

Due to the interaction of PlCYP5 with PlRPS15, we suspected that Δ*PlRPS15* phenocopies Δ*PlCYP5*. To validate this, we attempted to knockout the *PlRPS15* gene. Although we generated transformants in which expression of the *PlRPS15* gene was significantly decreased compared with that of the wildtype, we failed to obtain a complete knockout strain via conidia purification, implying that *PlRPS15* deletion leads to lethality. Nevertheless, we found that the expression of *PlCYP5* was also significantly decreased in the gene knockdown strain (Δ*PlRPS15_i*), and the Δ*PlRPS15_i* strain exhibited aberrant growth and development similar to that of the Δ*PlCYP5* strain (Supplementary Figure S7).

**Figure 6.**
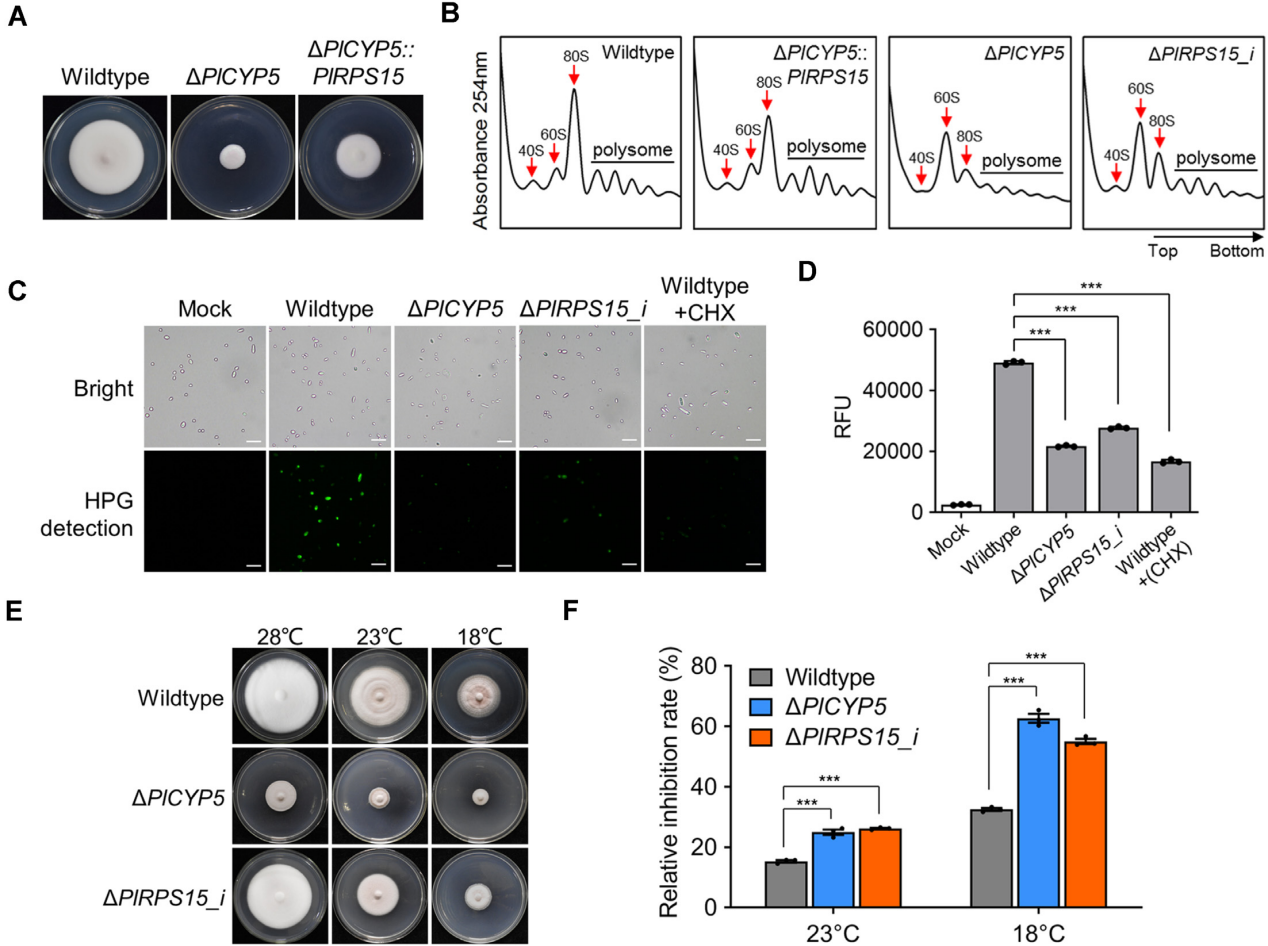
PlCYP5 and PlRPS15 mutants exhibit an impaired synthesis of ribosomes and new proteins. (**A**) Colony growth of wildtype, Δ*PlCYP5* and Δ*PlCYP5*::*PlRPS15* strains. (**B**) Polysome profiles of wildtype, Δ*PlCYP5*::*PlRPS15*, Δ*PlCYP5*, and Δ*PlRPS15_i*. Cell extracts were prepared after cycloheximide treatment and subjected to ultracentrifugal sedimentation on 7–47% sucrose density gradients. Absorbance was recorded at 254 nm. The peaks for 40S, 60S, 80S were indicated by red arrows. (**C**, **D**) The capacity of nascent protein synthesis in the wildtype, Δ*PlCYP5*, and Δ*PlRPS15_i* cells. An equal amount of blastospores of each strain was collected and subjected to the reaction using Click-iT^®^ protein reaction system. The nascent protein level was assessed by fluorescence microscope observation (C) and by determining signal intensity using a fluorescence microplate reader (D). Scale bar = 20 μm. (**E**) Colony growth of wildtype, Δ*PlCYP5* and Δ*PlRPS15_i* strains at normal temperature (28°C) and low temperature (23°C and 18°C). (**F**) Growth inhibition rate of wildtype, Δ*PlCYP5* and Δ*PlRPS15_i* strains at low temperature (23°C and 18°C) compared with normal temperature (28°C). The error bars indicate the SD of three replicates. *** denotes *P* < 0.001.

We then tested whether a high expression level of the *PlRPS15* gene suppressed the growth defect phenotype of Δ*PlCYP5*. We found that overexpression of the *PlRPS15* gene under a strong promoter (Δ*PlCYP5*::*PlRPS15*) partially rescued the growth defect of Δ*PlCYP5* (Figure 6A). The polysome profile of Δ*PlCYP5*::*PlRPS15* was similar to the wildtype (Figure 6B). Δ*PlCYP5* showed a reduced 40S peak and an increased 60S peak, resulting in blocking the formation of 80S and fewer polysome (Figure 6B). A similar abundance pattern of ribosomal subunits was also observed in the Δ*PlRPS15_i* cells (Figure 6B). These results suggested that PlCYP5 and PlRPS15 were involved in the same pathway. Since polysome represents the efficiency of protein synthesis, we compared the capacity of nascent protein synthesis between the wildtype and mutants by adding a detectable methionine substitute into the methionine-free medium. As expected, reduced fluorescence signal intensity was detected in the Δ*PlCYP5* and Δ*PlRPS15_i* cells (Figure 6C and D), representing fewer nascent proteins synthesized in Δ*PlRPS15_i* cells. Moreover, we tested the growth of the strains at low temperature and found that the growth inhibition rates of both Δ*PlCYP5* and Δ*PlRPS15_i* at 23°C and 18°C were significantly higher than those of the wildtype, suggesting that Δ*PlCYP5* and Δ*PlRPS15_i* could not synthesize sufficient ribosomes to compensate for the reduced translational ability at low temperature (Figure 6E and F). Hence, PlCYP5 played a role in ribosome biogenesis by interacting with PlRPS15, thereby regulating growth.

### PlCYP5 and PlRPS15 were required for ribosome biogenesis

**Figure 7.**
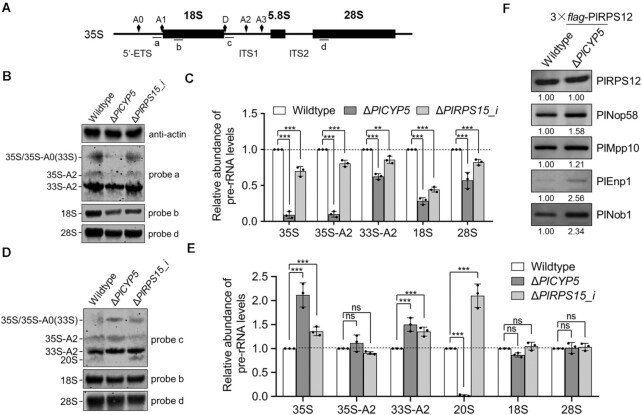
PlCYP5 contributes to ribosome biogenesis. (**A**) Schematic diagram of 35S pre-rRNA of *P*. *lilacinum*. The rRNA cleavage sites and the binding sites of probes used for northern blotting are indicated. ETS: external transcribed space. ITS1 and 2: Internal transcribed space 1 and 2. (**B**, **C**) Northern blot (B) and quantitative analyses (C) of pre-rRNA and mature rRNA in wildtype, Δ*PlCYP5* and Δ*PlRPS15_i* corresponding to equal biomass. The strains were cultured at 28°C to the vegetative growth stage. Total RNAs of the same biomass of the strains were isolated, separated, and transferred to a nylon membrane. The pre-rRNA and mature rRNA were detected using the following probes: probe a for detection of 35S/35S-A0, 35S-A2 and 33S-A2 pre-rRNA, probe c for detection of 35S/35S-A0, 35S-A2, 33S-A2, and 20S pre-rRNA, probe b for detection of 18S rRNA, probe d for detection of 28S rRNA. The abundance of pre-rRNA and mature rRNA in Δ*PlCYP5* and Δ*PlRPS15_i* cells is presented relative to wildtype cells from three independent replicates. (**D**, **E**) Northern blot (D) and quantitative analyses (E) of pre-rRNA and mature rRNA in wildtype, Δ*PlCYP5* and Δ*PlRPS15_i* corresponding to the same loading amount of total RNA (7 μg). The abundance of pre-rRNA and mature rRNA in Δ*PlCYP5* and Δ*PlRPS15_i* cells is presented relative to wildtype cells from three independent replicates. (**F**) The Enhanced association of assembly factors with pre-ribosomes in the absence of PlCYP5. 3 × *flag*-PlRPS12 was purified from the nuclear extracts of wildtype and Δ*PlCYP5* via anti-*flag* affinity sepharose. The purified samples were analyzed by western blot to detect the presence of PlRPS12, PlNop58, PlMpp10, PlEnp1, and PlNob1. Signals were quantified by ImageQuant TL (GE Healthcare). The error bars indicate the SD of three replicates. ns denotes *P* > 0.05, ** denotes *P* < 0.01, *** denotes *P* < 0.001.

To elucidate the mechanism underlying the regulation of ribosome biogenesis by PlCYP5, we designed specific probes targeting pre-rRNA and used them to detect pre-rRNA abundance in total RNA samples extracted from the wildtype, Δ*PlCYP5* and Δ*PlRPS15_i* cells (Figure 7A). Due to the difference in growth rates among these strains, we first compared the RNA samples from the equal biomass of these strains. It showed that the abundance of mature 25S and 18S rRNAs in Δ*PlCYP5* were significantly less than those of the wildtype, which was caused by the reduced accumulation of 35S pre-rRNA (Figure 7B and C). To explore the specific cleavage steps affected by PlCYP5 and PlRPS15, we further examined the levels of pre-rRNA processing intermediates. Compared with the level of RNAs in the wildtype, increased accumulation of 35S pre-rRNA was observed in both Δ*PlCYP5* and Δ*PlRPS15_i*, representing a delayed 35S pre-rRNA cleavage. Moreover, in the Δ*PlCYP5* cells, the abundance of intermediate products, 35S-A2 and 33S-A2 that were obtained by cleavage at A2 sites of 35S pre-rRNA, increased (Figure 7A, D, and E). This associated with a decreased level of 20S rRNA that was generated by the cleavage at the A1 site of 33S-A2 (Figure 7A, D and E).

The role of PlCYP5 in pre-rRNA cleavage implied that PlCYP5 also affected the assembly of pre-ribosome. To test this, we purified 3 × *flag*-PlRPS12 and its associated proteins from the nuclear extracts of the wildtype and Δ*PlCYP5* strains and detected the abundance of pre-ribosomal assembly factors, normalized to the level of PlRPS12. Compared with the wildtype, the 90S and pre-40S assembly factors, including PlNop58, PlMpp10, and PlEnp1, were more abundant in Δ*PlCYP5*, suggesting an enhanced association between these assembly factors and pre-ribosomes in Δ*PlCYP5* (Figure 7F). Since the assembly of pre-ribosomes was a cooperative process of the binding and release of assembly factors, this enhanced association suggested that PlCYP5 regulated the assembly of pre-ribosomes. In addition, the abundance of PlNob1, whose homolog in *S. cerevisiae* was responsible for the 20S rRNA cleavage after being exported from the nucleus together with the pre-40S, also increased in Δ*PlCYP5* (Figure 7F). Together with the reduced 20S production in Δ*PlCYP5* (Figure 7D), PlCYP5 might have also affected the pre-40S nuclear export. Thus, PlCYP5 played a role in coordinating the smooth progress of ribosome biogenesis.

### PlCYP5 protected PlRPS15 from degradation and aggregation independently of its PPIase activity

**Figure 8.**
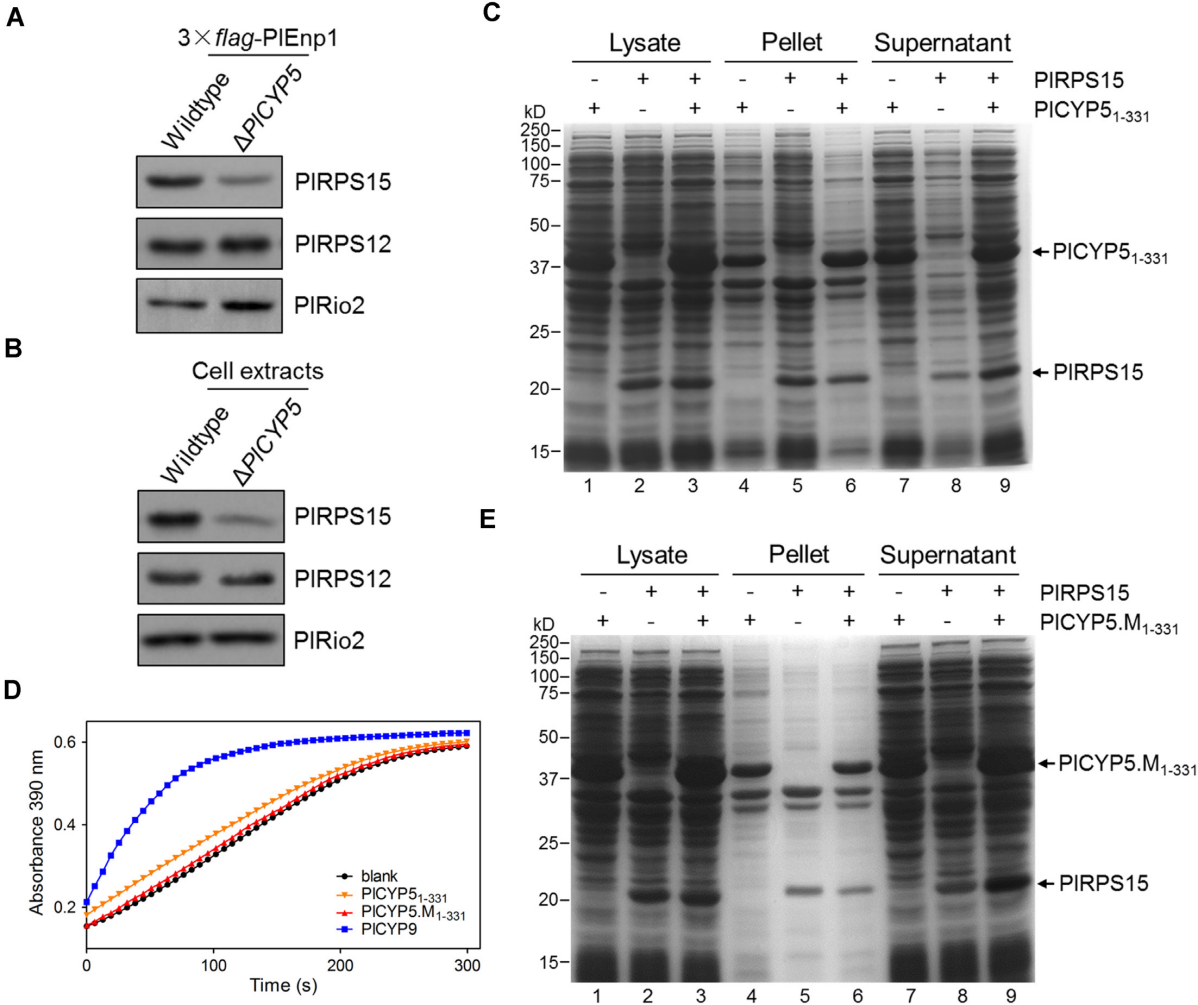
PlCYP5 possesses a PPIase independent chaperone function for PlRPS15. (**A**) PlCYP5 facilities the recruitment of PlRPS15 to pre-ribosome. 3 × *flag*-PlEnp1 was isolated from wildtype and Δ*PlCYP5* cells and purified via anti-*flag* affinity sepharose. Proteins were separated by SDS-PAGE, followed by western blot to detect the presence of PlRPS15, PlRPS12, and PlRio2. (**B**) PlCYP5 maintains the protein level of PlRPS15 in cells. Total proteins were extracted from wildtype and Δ*PlCYP5* cells and analyzed by western blot to detect the presence of PlRPS15, PlRPS12 and PlRio2. (**C**) Solubility test of PlCYP5_1–331_ and PlRPS15 in bacteria. PlRPS15 was either expressed alone or co-expressed with PlCYP5_1-331_ in *E. coli*. Cells were lysed by sonication and lysates were centrifuged at 12 000 rpm to separate pellet and supernatant. Samples from the lysate, pellet and supernatant were analyzed by SDS-PAGE and Coomassie blue staining. (**D**) PPIase assay of PlCYP5_1-331_ and its mutation (PlCYP5.M_1–331_). PlCYP5_1-331_, PlCYP5.M_1-331_ and PlCYP9 were expressed and purified from *E. coli*, respectively. Equal amounts of proteins were used for the PPIase assay. Equal volume of reaction buffer was used to replace protein as blank control. (**E**) Solubility test of PlCYP5.M_1–331_ and PlRPS15 in bacteria.

Because PlCYP5 was not associated with the pre-ribosome, it was likely that PlCYP5 acted as a molecular chaperone to support the transfer of PlRPS15 to the pre-ribosome. To test this hypothesis, we purified 3 × *flag*-PlEnp1 and its associated proteins from cell extracts of the wildtype and Δ*PlCYP5* strains. Compared with the wildtype, less PlRPS15 was co-purified in Δ*PlCYP5* (Figure 8A). Similarly, the protein level of PlRPS15 was reduced in the total cell extracts of Δ*PlCYP5* (Figure 8B), suggesting that the decreased association of PlRPS15 with pre-ribosomes was due to the decreased PlRPS15 protein level in Δ*PlCYP5*, suggesting that PlCYP5 facilitated the recruitment of PlRPS15 to the pre-ribosomes by maintaining PlRPS15 protein accumulation.

Like other RPs, PlRPS15 expressed in *E. coli* was prone to aggregation as most proteins were present in the pellet (Figure 8C, lanes 2, 5 and 8). To determine whether PlCYP5 prevented the aggregation of PlRPS15, PlCYP5 was co-expressed with *PlRPS15* in *E. coli*. However, when the full-length sequence of PlCYP5 was expressed, only a small fraction of PlCYP5 was soluble (Figure 5B). Because the CR of PlCYP5 was expressed with low solubility, and since it did not interact with PlRPS15 (Figure 5D and E), we chose to express the CR-lacking PlCYP5 (PlCYP5_1–331_). We found a noticeable increase in the amount of soluble proteins (Figure 8C, lanes 1, 4 and 7). Notably, we found that a large proportion of PlRPS15 became more soluble when PlCYP5_1-331_ was co-expressed (Figure 8C, lanes 3, 6 and 9). These results demonstrated that PlCYP5 increased the solubility of PlRPS15.

The CLD of PlCYP5 was a primary domain for the PlCYP5-PlRPS15 interaction (Figure 5D). This prompted us to investigate whether the PPIase activity contributed to the protection of PlRPS15. We previously revealed that most amino acids required for the PPIase activity on the CLD of PlCYP5 were not conserved except for the Q^63^ amino acid (41). Indeed, PlCYP5_1–331_ exhibited weaker PPIase activity than PlCYP9 which was the homolog of human CYPA (Figure 8D). We then generated a mutant variant of PlCYP5 (PlCYP5.M_1–331_), which lost its PPIase activity due to the point mutation at Q^63^ (Figure 8D). The co-expression test showed that PlCYP5.M_1–331_ increased the solubility of PlRPS15 as efficiently as PlCYP5_1–331_ (Figure 8E), suggesting that PPIase activity of PlCYP5 is not required for its anti-aggregation function.

### PlCYP5 co-translationally interacted with the N-terminal extension of PlRPS15

**Figure 9.**
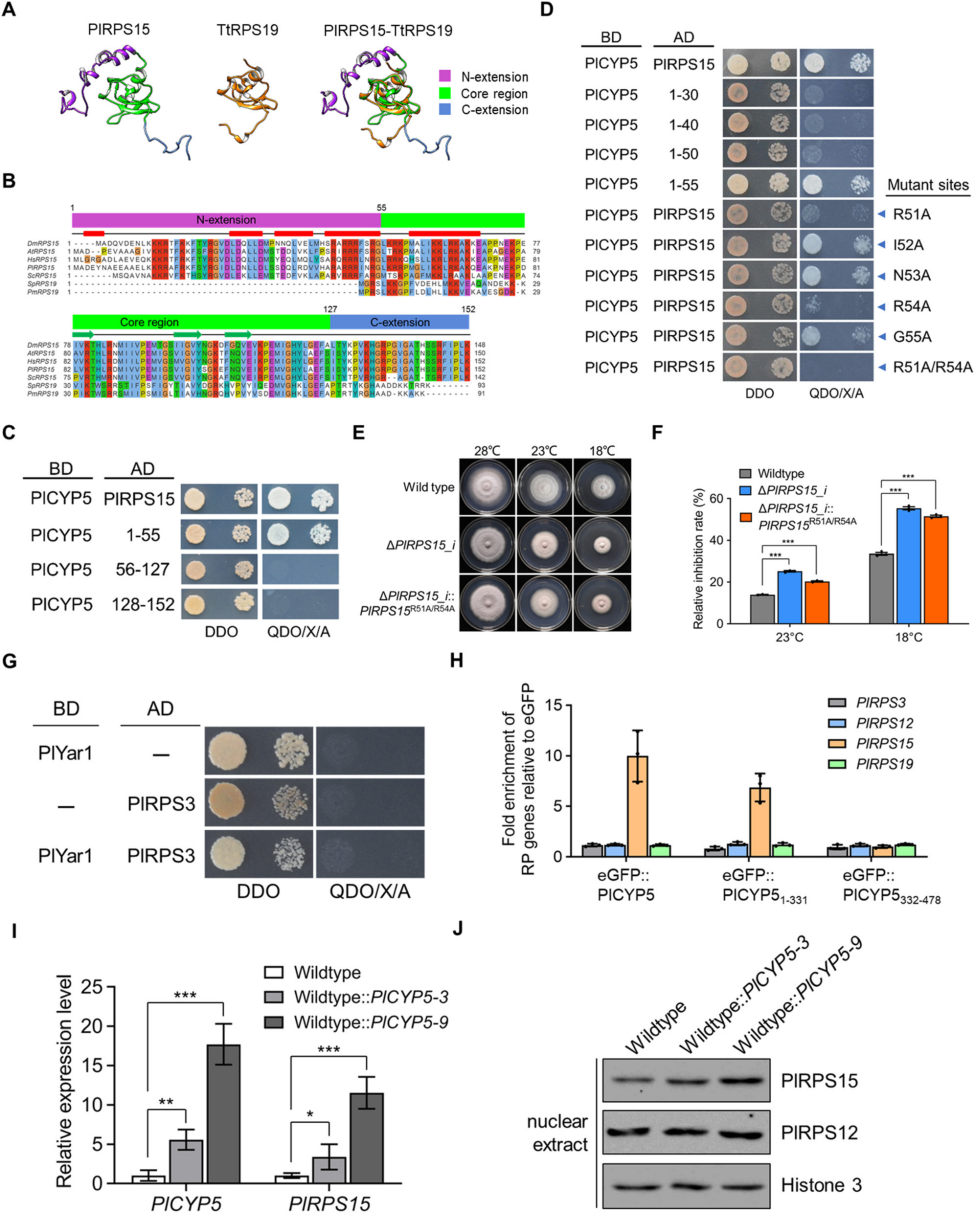
PlCYP5 binds to the N-terminal extension of PlRPS15. (**A**) Protein structure match between PlRPS15 of *Purpureocillium lilacinum* and TtRPS19 of *Thermus thermophiles*. Protein spatial structure of PlRPS15 was predicted by ITASSER server and the crystal structure of TtRPS19 was downloaded from PDB (PDB id: 1QKF). (**B**) Multiple sequence alignment of uS19 family (including S15 of eukaryote and S19 of bacteria). The N-terminal extension, C-terminal extension, and conserved core region were indicated above the sequences. Dm: *Drosophila melanogaster*, At: *Arabidopsis thaliana*, Hs: *Homo sapiens*, Pl: *P. lilacinum*, Sc: *Saccharomyces cerevisiae*, Sp: *Streptococcus pneumonia*, Pm: *Pasteurella multocida*. (**C**) Y2H interaction between PlCYP5 and truncated PlRPS15. PlRPS15 was divided into three truncations including the N-terminal (1–55), core region (56-127), and C-terminal (128–152). (**D**) Y2H interaction between PlCYP5 and the truncated N-terminus of PlRPS15. The blue arrowheads indicate the residues mutated in PlRPS15. (**E**) Colony growth of wildtype, Δ*PlRPS15* and Δ*PlRPS15*::*PlRPS15*^R51A/R54A^ strains at normal temperature (28°C) and low temperature (23°C and 18°C). (**F**) Growth inhibition rate of wildtype, Δ*PlRPS15* and Δ*PlRPS15*::*PlRPS15*^R51A/R54A^ strains at low temperature (23°C and 18°C) compared with normal temperature (28°C). (**G**) Y2H interaction between PlYar1 and PlRPS3. (**H**) Relative mRNA enrichment for the indicated RP mRNAs in eGFP::PlCYP5, eGFP::PlCYP5_1–331_, and eGFP::PlCYP5_332-478_ purifications versus the control eGFP purification. (**I**) Relative expression levels of *PlCYP5* and *PlRPS15* genes in the *PlCYP5* overexpression strains versus the wildtype. Two *PlCYP5* overexpression strains were obtained, with low expression level (Wildtype::*PlCYP5-3*) and high expression level (Wildtype::*PlCYP5-9*). RT-qPCR was used to detect the mRNA level. (**J**) PlCYP5 facilitates the nuclear localization of PlRPS15. Nuclear proteins were extracted from the wildtype and *PlCYP5* gene overexpression strains and analyzed by western blot to detect the presence of PlRPS15 and PlRPS12. The antibody histone 3 was used to quantify the blots. The error bars indicate the SD of three replicates. *** denotes *P* < 0.001.

To gain more insights into the PlCYP5-PlRPS15 interaction, we investigated the binding site in PlRPS15. PlRPS15 belongs to the uS19 family, which includes the RPS15 family in eukaryotes and the RPS19 family in prokaryotes. Hence, we first compared amino acid sequences of these two families. The protein structure of PlRPS15 was also predicted and matched with the crystal structure of RPS19 from *Thermus thermophilus* (PDB id: 1QKF). There was a conserved core region with a similar structure for both proteins (Figure 9A). Apart from the core region, PlRPS15 harbored a longer N-terminal and a shorter C-terminal extension compared with TtRPS19 (Figure 9A). Sequence alignment further identified 56–127 aa of PlRPS15 as the core region, and 1–55 aa and 128–152 aa as the N-terminal and C-terminal extension (Figure 9B). Based on the alignment, we divided PlRPS15 into these three parts. Each part was used for the Y2H assay with PlCYP5. The results showed that the N-terminal extension interacted with PlCYP5, while both the core region and the C-terminal extension did not (Figure 9C).

We further tested different length of the N-terminal extension for interaction with PlCYP5 and found a robust interaction with PlCYP5 when amino acid residues 51–55 (R^51^I^52^N^53^R^54^G^55^) were present (Figure 9D). Point mutation of these amino acids to alanine (A) was introduced to investigate important residues contributing to the interaction. The single mutation of I52A, N53A or G55A did not affect the interaction. In contrast, the mutation of R51A or R54A attenuated the protein interaction, and the double-point mutations R51A/R54A caused a complete loss of the interaction (Figure 9D). To investigate the physiological significance of these amino acid residues that are required for the interaction with PlCYP5, we complemented Δ*PlRPS15_i* with *PlRPS15*^R51A/R54A^ and determined the growth phenotype at low temperatures. In agreement with the Y2H results, *PlRPS15*^R51A/R54A^ could not rescue the growth retardation phenotype of Δ*PlRPS15_i* at low temperatures (Figure 9E and F). In addition, we overexpressed the wildtype *PlRPS15* and *PlRPS15*^R51A/R54A^ in the wildtype and Δ*PlCYP5* strains and obtained transformants with low or high expression level, respectively (Supplementary Figure S8A). Then, the polysome profiles of wildtype, Δ*PlCYP5*, and these overexpression strains were analyzed. In the wildtype, there was no obvious difference in polysome profile among the wildtype and strains expressing *PlRPS15*^R51A/R54A^ at the low and high expression level (Supplementary Figure S8B, top row). In contrast, low and high *PlRPS15* expression in Δ*PlCYP5*, either wildtype *PlRPS15* or *PlRPS15*^R51A/R54A^, produced distinct results. Low expression of wildtype *PlRPS15* in Δ*PlCYP5* partially restored the polysome profile. However, the strain with low expression of *PlRPS15*^R51A/R54A^ exhibited the abnormal 60S/40S ratio as in Δ*PlCYP5* (Supplementary Figure S8B, bottom row). Δ*PlCYP5* with high expression of wildtype *PlRPS15* or *PlRPS15*^R51A/R54A^ could form prominent 80S peaks, but the 40S and 80S abundance in the strain expressing *PlRPS15*^R51A/R54A^ was lower than that of the strain expressing wildtype *PlRPS15* (Supplementary Figure S8B, bottom row). These results suggest that PlCYP5 excert its function via physical interaction with PlRPS15 for the formation and stabilization of 40S.

PlCYP5 might be associated with PlRPS15 in a co-translational manner as PlCYP5 bound to the eukaryote-specific N-terminus of PlRPS15 like other RP dedicated chaperones, such as Yar1 (15). If PlCYP5 co-translationally interacted with PlRPS15, one would expect that PlRPS15 mRNA would be co-purified with PlCYP5. To explore this hypothesis, we purified PlCYP5 after translation inhibition by cycloheximide, and RNA was extracted from the purified samples. The mRNA levels of *PlRPS15* as well as *PlRPS3*, *PlRPS12* and *PlRPS19* were analyzed by RT-qPCR. Prior to this, we attempted to verify the interaction between PlYar1 and PlRPS3, two proteins whose homologs in yeast have been reported to bind co-translationally, so that this could be used as a reference for gene enrichment. However, we did not observe the interaction between Yar1 and RPS3 of *P*. *lilacinum* in the Y2H assay (Figure 9G). Nevertheless, PlCYP5 and PlCYP5_1-331_ were co-purified with *PlRPS15*, but not *PlRPS3*, *PlRPS12* and *PlRPS19* (Figure 9H). To obtain more evidence, we investigated the effect of increased *PlCYP5* on the level of *PlRPS15* and found that the expression of *PlRPS15* was also higher in the strain with high *PlCYP5* expression (Figure 9I). Moreover, high expression of *PlCYP5* induced a more PlRPS15 protein level in the nucleus (Figure 9J), suggesting that PlCYP5 facilitated the nuclear import of PlRPS15. Collectively, we concluded that PlCYP5 co-translationally interacted with the N-terminal extension of PlRPS15.

### Interaction between the RRM-containing CYPs and RPS15 family was unique in the filamentous fungi

**Figure 10.**
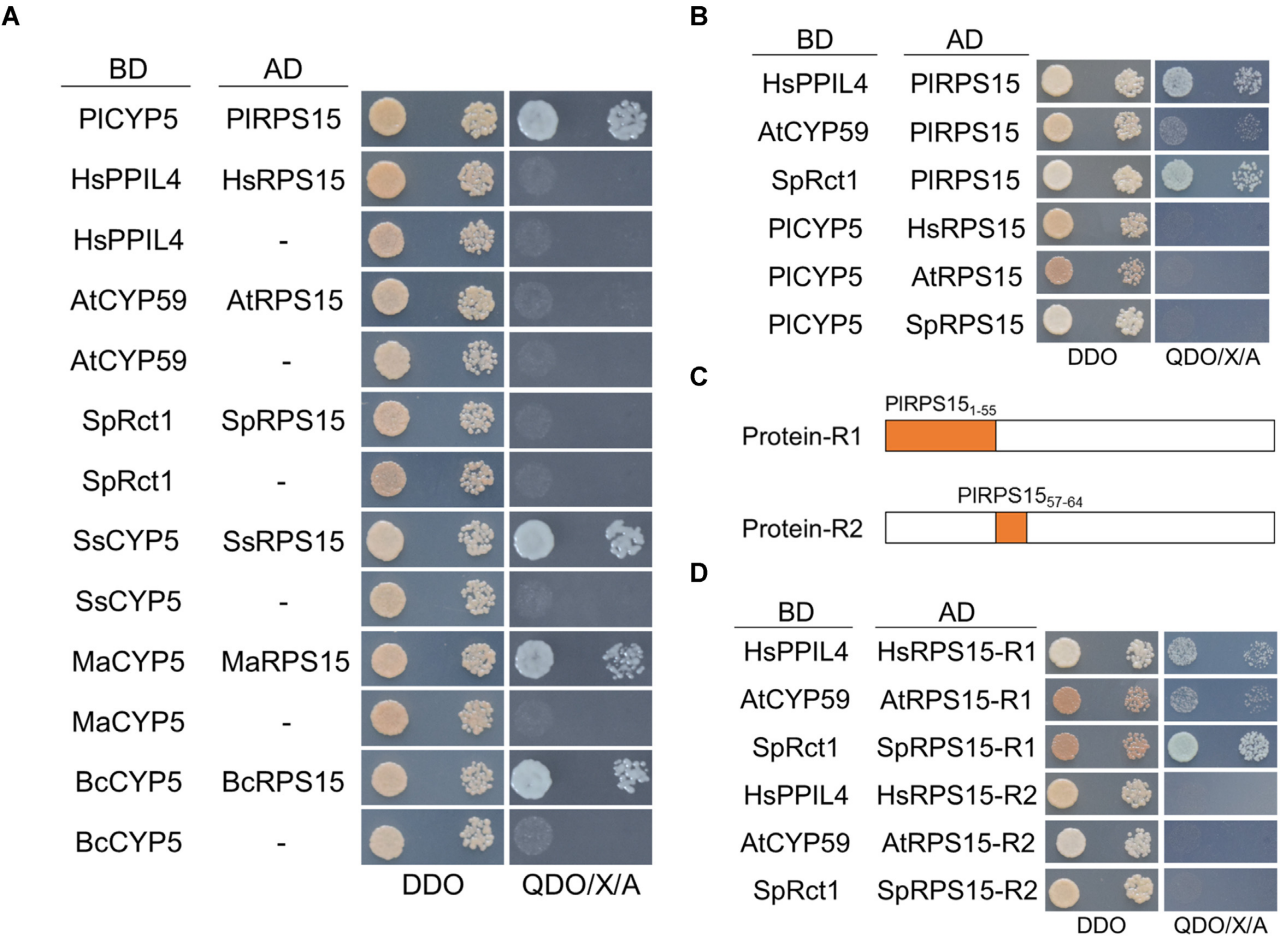
N-terminal extension of the RPS15 family contributes to the interaction with RRM-containing CYPs in filamentous fungi. (**A**) Y2H interaction between RRM-containing CYPs and RPS15 family proteins in different species. The RRM-containing CYPs were cloned into vector pGBKT7 (BD) and RPS15 family proteins were cloned into vector pGADT7 (AD). Hs: *Homo sapiens*, At: *Arabidopsis thaliana*, Sp: *Schizosaccharomyces pombe*, Ss: *Sclerotinia sclerotiorum*, Ma: *Metarhizium anisopliae*, Bc: *Botrytis cinerea*. (**B**) Cross Y2H interaction between RRM-containing CYPs and RPS15 family in different species. (**C**) Schematic diagram of sequence replacement of the RPS15 family using the corresponding sequence of PlRPS15. (**D**) Y2H interaction between RRM-containing CYPs and recombinant RPS15 proteins in different species.

Although the PlCYP5 homolog was absent in *S. cerevisiae* and other Saccharomycotina fungi, it has been identified in other species, such as HsPPIL4 in *Homo sapiens*, AtCYP59 in *Arabidopsis thaniana*, and SpRct1 in *Schizosaccharomyces pombe*. Therefore, we analyzed the interactions between the PlCYP5 and PlRPS15 homologs from these species. Not surprisingly, the interactions were absent for *H*. *sapiens*, *A*. *thaniana* and *S*. *pombe* homologs, but were present for the homologs from filamentous fungi, including *Sclerotinia sclerotiorum*, *Metarhizium anisopliae* and *Botrytis cinerea* (Figure 10A). To determine the reason for the distinct interactions, Y2H assay was conducted to test cross-species interactions. The results showed that HsPPIL4, AtCYP59 and SpRct1 interacted with PlRPS15 with different intensities, while PlCYP5 did not interact with any RPS15 from these species. This suggested that the difference in the RPS15 sequences determined whether PlCYP5 and PlRPS15 homologs interact (Figure 10B). Sequence alignment of the PRS15 family proteins revealed two variable regions. One was the N-terminal extension of amino acids 1–55, and the other was amino acids 57–64 (Supplementary Figure S9). Thus, each of these two regions of HsRPS15, AtRPS15 and SpRPS15 was replaced with the corresponding sequences of PlRPS15 (Figure 10C). Y2H assay was then conducted. Notably, HsRPS15, AtRPS15 and SpRPS15 with the replacement of the N-terminal extension (1–55 aa) from PlRPS15 interacted with HsPPIL4, AtCYP59 and SpRct1 while those with the replacement of amino acids 57–64 did not (Figure 10D). These results indicated that the difference in N-terminal sequences of RPS15 between filamentous fungi and other species is a key factor for the interaction of PlCYP5-PlRPS15 homologs.

## DISCUSSION

**Figure 11.**
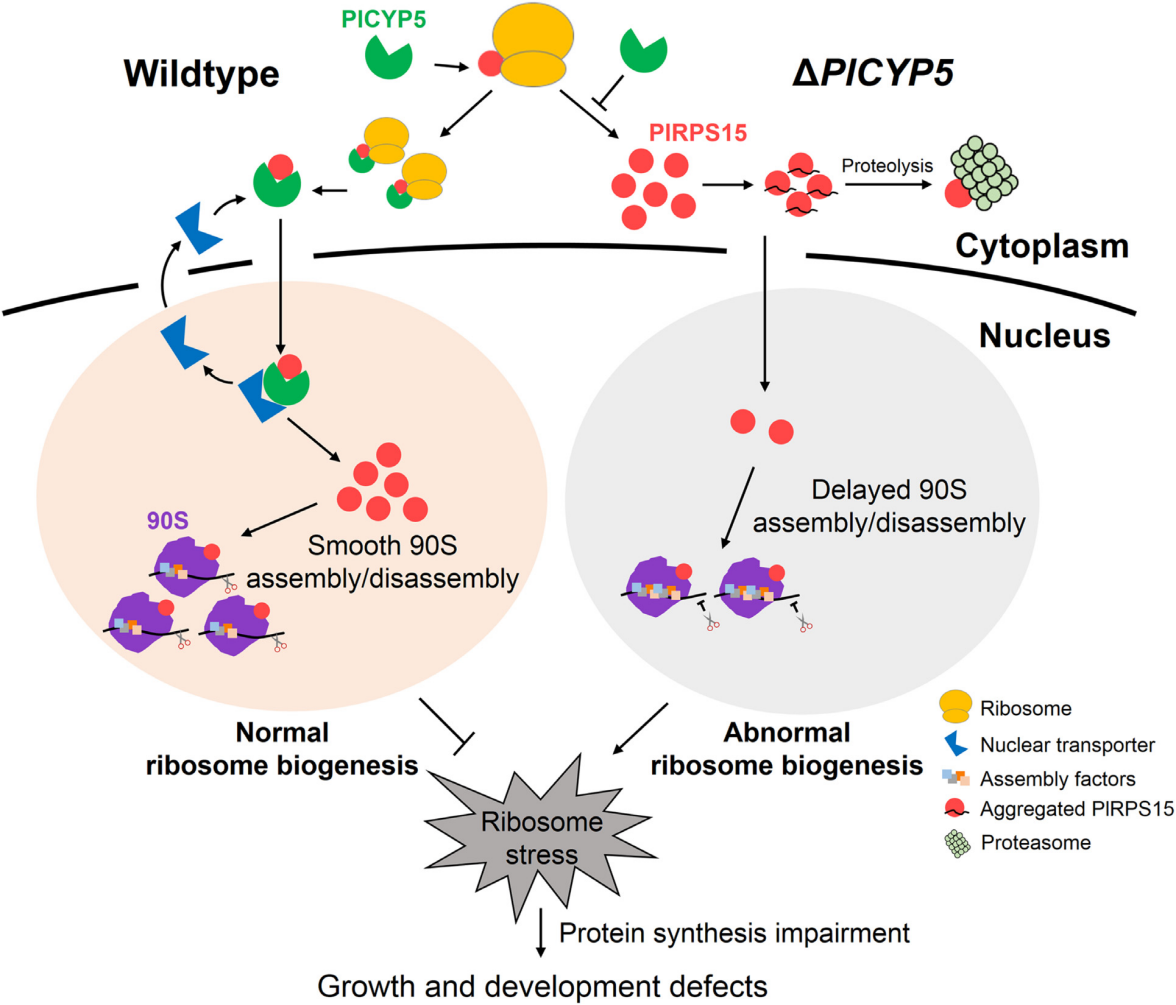
A working model of PlCYP5-PlRPS15 interaction contributing to ribosome biogenesis in *P*. *lilacinum*.

Previously, we found that *PlCYP5* gene is expressed during the nematode egg parasitism and upon exposure to abiotic stressors (41). These results, together with the pleiotropic phenotype of the *PlCYP5* deletion mutant, implied that *PlCYP5* had an essential function for multiple biological processes. In this study, we identified PlCYP5 as a physical interaction partner of PlRPS15 (uS19) and a factor that regulated ribosome biogenesis. Additional evidence further revealed that PlCYP5 acted as a dedicated chaperone for PlRPS15. First, PlCYP5 was not associated with the pre-ribosomal complex. Second, RPs easily aggregated due to positively charged amino acids in the extension. A common mechanism by which the dedicated RP chaperones protected their target from aggregation is to cover these extensions through co-translational interaction. For example, the dedicated chaperone Yar1 interacted with the N-terminus of RPS3 to maintain its solubility (20,21). Sqt1 and Rrb1 interacted with the N-terminal region of RPL10 and RPL3, respectively (15). Tsr4 bound to the N-terminal 42 amino acids of RPS2 (49). Likewise, we found that PlCYP5 (p*I* = 4.67) bound co-translationally to the N-terminal extension of PlRPS15 (p*I* = 9.80) and exhibited an anti-aggregation function to maintain its solubility. Third, PlCYP5 had a feeble enzyme activity, which was consistent with our finding that multiple residues in PlCYP5 required for PPIase activity were not conserved (41). In addition, even without the PPIase activity, PlCYP5 still interacted with and protected PlRPS15 from aggregation, indicating that the PlCYP5 PPIase activity was not responsible for the interaction with PlRPS15. Collectively, we propose a working model for the PlCYP5–PlRPS15 interaction in ribosome biogenesis (Figure 11). In wildtype cells, PlCYP5 is co-tranlationally interacted with PlRPS15 in the cytoplasm, and the complex is transported into the nucleus. The protection of PlRPS15 provided by PlCYP5 allows PlRPS15 to be transferred to the ribosomal assembly site, where PlRPS15 forms the 90S with proper pre-rRNA processing and assembly factors binding/release. However, in Δ*PlCYP5* cells, the loss of PlCYP5-PlRPS15 interaction leads to the non-specific aggregation and degradation of PlRPS15, resulting in abnormal ribosome biogenesis, which is manifested by the retarded pre-rRNA processing and 90S assembly. Hence, the Δ*PlCYP5* cells exhibit a ribosomal stress, which ultimately compromises normal cell growth and development.

The *PlCYP5* mutant exhibited various growth and development abnormalities, one of which was the hyperbranching under a slow growth rate (Figure 3A). Fungal cells possess an internal homeostatic system to maintain a certain branch density under diverse growth rates caused by external factors (e.g. changes in temperature and growth nutrient) (50,51). However, the branching homeostasis was perturbed in some gene knockout mutants, resulting in branch density changes (51). In this case, our finding suggests that *PlCYP5* might play a role in the branching homeostasis system. We also found that the protein content in Δ*PlCYP5* fermentation was reduced than that in the wildtype, and the nematocidal activity of Δ*PlCYP5* fermentation was significantly decreased after incubation with nematodes for 3 days (Figure 3E–G). This raised a question whether the nematocidal activity of Δ*PlCYP5* fermentation could reach to the level of wildtype when incubation more than 3 days. To solve this, we measured the nematocidal activity lasting to 7 days, however, it showed no significant difference from the result of 3 days. This indicated that the lack of nematocidal activity of Δ*PlCYP5* fermentation was due to the loss of key nematocidal proteins rather than just the reduced protein content.

Transcriptome analysis revealed that a large number of ribosome-related genes were differentially expressed in Δ*PlCYP5*, which was consistent with a previous study that discovered a transcriptional feedback response to ribosome biogenesis impairment (52). Surprisingly, we found the genes encoding ribosomal assembly factors were generally up-regulated in Δ*PlCYP5* compared with those in wildtype (Figure 3B). This was reminiscent of the phenotype found in a range of RPS (RPs of 40S) mutants (53), suggesting that Δ*PlCYP5* might exhibit similar gene expression signatures as those of RPS mutants. However, a common phenotype of RPS mutants was up-regulation of the RP genes (53), but we found that all RP genes were down-regulated in Δ*PlCYP5* (Figure 3E). It is probably because the RRM-containing CYPs, including PlCYP5, play an extra role in the direct regulation of the transcription process (42,43). In addition, for the discrepancy in gene expression between the RPs and ribosomal assembly factors responding to ribosome biogenesis impairment, one explanation in yeast was that these two sets of genes were controlled by distinct transcription factors. Although homologous genes of some critical transcription factors for RPs and ribosomal assembly factors in yeast were not found in *P*. *lilacinum*, such as Rap1 and Stb3 (54,55), those transcriptional factors with identified homologous genes had the same expression patterns with their target genes. For example, the transcription factor *Hmo1* was down-regulated as the Hmo1-regulated RP genes, while the *Dot6* and *Spt6*, which drove the expression of ribosomal assembly factors, were up-regulated (54,56). This suggests that these transcription factors that regulate the expression of ribosome-related genes in yeast have a similar function in filamentous fungi.

Currently, the nuclear import mechanism of numerous RPs, including RPS15 (uS19), remains unknown. The observation that PlCYP5 co-translationally interacted with PlRPS15 manifested that PlRPS15 was transported into the nucleus together with PlCYP5. Under this premise, we attempted to explore the mechanism of PlCYP5-PlRPS15 nuclear transport. RPs enter the nucleus with the assistance of importins. In *S. cerevisiae*, a number of importins that transport RPs into the nucleus have been identified, including Kap104, Kap108, Kap121 and Kap123 (19,22,57). PlCYP5 has been proved to possess NLSs, and PlRPS15 was also predicted to contain an NLS at the N-terminal extension. Hence, we used Y2H assay to investigate the interactions of these importins with PlCYP5 and PlRPS15 in *P. lilacinum*, respectively. However, the result demonstrated that none of the homologs of the yeast importins in *P. lilacinum* interacted with either PlRPS15 or PlCYP5. A possible reason was that unidentified importins mediate nuclear import of PlRPS15 or that other mechanisms than importin assist RPs in entering the nucleus in filamentous fungi. Nonetheless, it suggested that the charged N-terminal extension of PlRPS15 needed to be covered to prevent aggregation either by PlCYP5 or by as-yet-undiscovered nuclear transporter.

Overexpression of *PlRPS15* in the *PlCYP5* mutant did not completely restore its slow-growth phenotype. It might be due to the other functions of PlCYP5. Aside from the CLD, PlCYP5 contained another functional domain, the RRM, which was not involved in the interaction with PlRPS15 but was well known for the function of binding RNA molecules with a wide range of specificities and affinities (58,59). For example, the homolog of PlCYP5 in *A*. *thaliana* (AtCYP59) regulated the RNAP II transcription and pre-mRNA processing and had the capacity to bind RNA through its RRM (42,60). Similarly, the PlCYP5 homolog, SpRct1, in *S*. *pombe* also affected the RNAP II transcription (43). Subsequently, SpRct1 was proved to function in the RNAi pathway and its RRM was required for the siRNA biogenesis (61). Our study showed that PlCYP5 had the same nuclear localization with AtCYP59 and SpRct1. Moreover, the transcriptome analysis identified a large number of genes differentially expressed in the PlCYP5 loss-of-function mutant compared with the wildtype. It suggested that PlCYP5 might also perform a similar function in transcriptional regulation through its RRM. Therefore, overexpression of *PlRPS15* did not compensate for the loss of PlCYP5.

A large number of assembly factors have been identified and proven essential for ribosome biogenesis since they are recruited to pre-rRNA at specific stages to assist in the pre-rRNA processing (62–64). In contrast to assembly factors, dedicated RP chaperones are not associated with the pre-ribosomal particles. Nevertheless, biological cells lacking these RP chaperones also exhibited abnormal ribosome biogenesis, such as blocked pre-rRNA processing and pre-40S/pre-60S nuclear export (2,20,23). It was probably because the absence of a dedicated chaperone usually resulted in the instability of its target protein. Indeed, we found the reduced amount of PlRPS15 in the *PlCYP5* knockout mutant cells and interference with the *PlRPS15* gene caused a similar cellular ribosomal stress phenotype as the *PlCYP5* mutant. Thus, our findings highlight the importance of dedicated chaperones for RP proteins in ribosome biogenesis.

## DATA AVAILABILITY

RNA-seq data have been deposited in the Gene Expression Omnibus (GEO) under accession number GSE179712.

## Supplementary Material

gkab1102_Supplemental_Files
