# Supplementary material for: Cyclophilin acts as a ribosome biogenesis factor by chaperoning the ribosomal protein (PlRPS15) in filamentous fungi
Source: Nucleic Acids Res. 2021 Nov 18;49(21):12358–76. doi: 10.1093/nar/gkab1102 (PMC8643696; doi:10.1093/nar/gkab1102)
Supplement: gkab1102_Supplemental_Files [file gkab1102_supplemental_files.zip › Supplementary Figures.pdf]

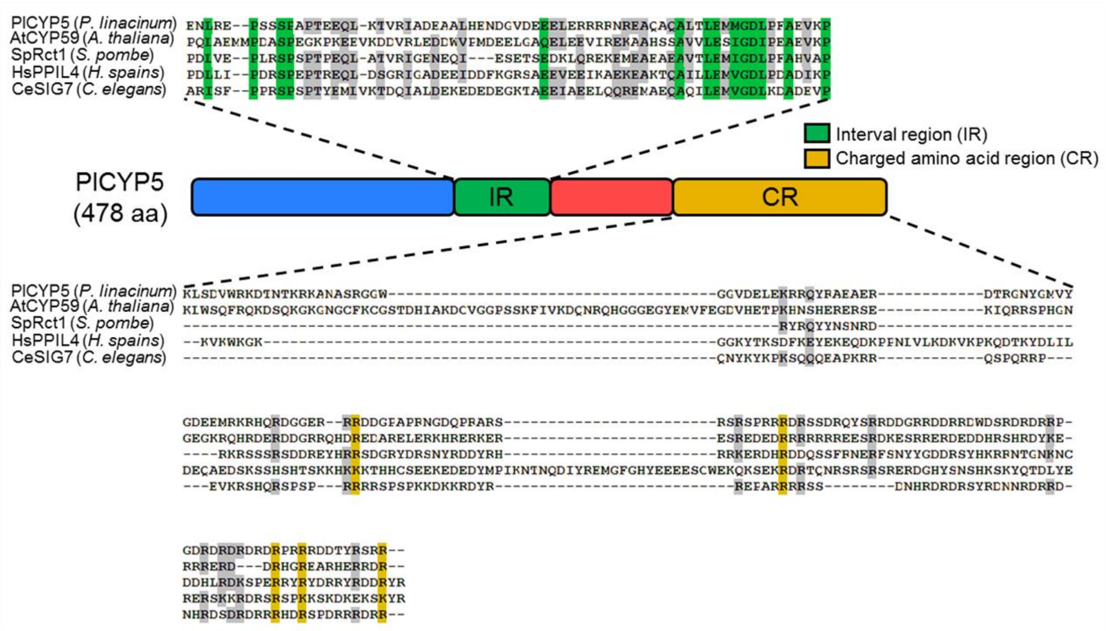

Figure S1. Multiple sequence alignment of the IR and CR of RRM-containing CYPs from different species. Amino acids with different colored background indicate different conservation. Pl: *Purpureocillium lilacinum*, At: *Arabidopsis thaliana*, Sp: *Schizosaccharomyces pombe*, Hs: *Homo sapiens*, Ce: *Caenorhabditis elegans*.

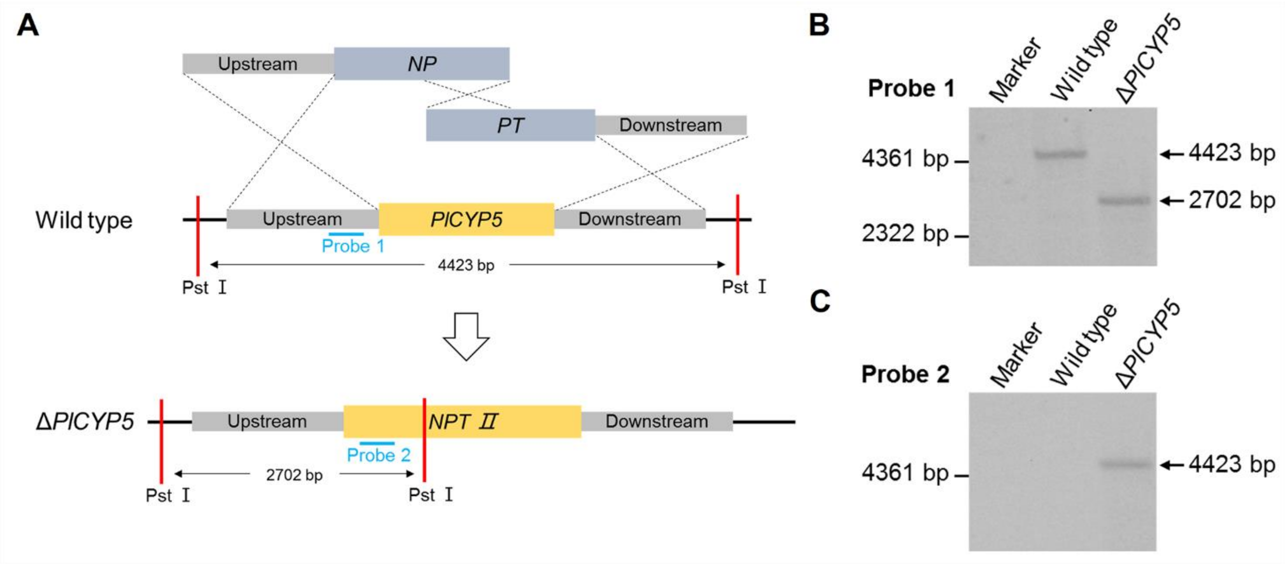

Figure S2. Single copy validation of *PlCYP5* gene mutant by southern blot. **(A)** Schematic diagram of gene knockout strategy and probes design. The genomic DNA of wildtype and Δ*PlCYP5* was digested with Pst I. Probe 1 was designed on the upstream of *PlCYP5* and probe 2 was designed on the *NPT II* gene. *NPT II*: G418 sulfate resistance gene. **(B and C)** Southern blot analysis of the wildtype and Δ*PlCYP5* strains using probe 1 **(B)** and probe 2 **(C)**.

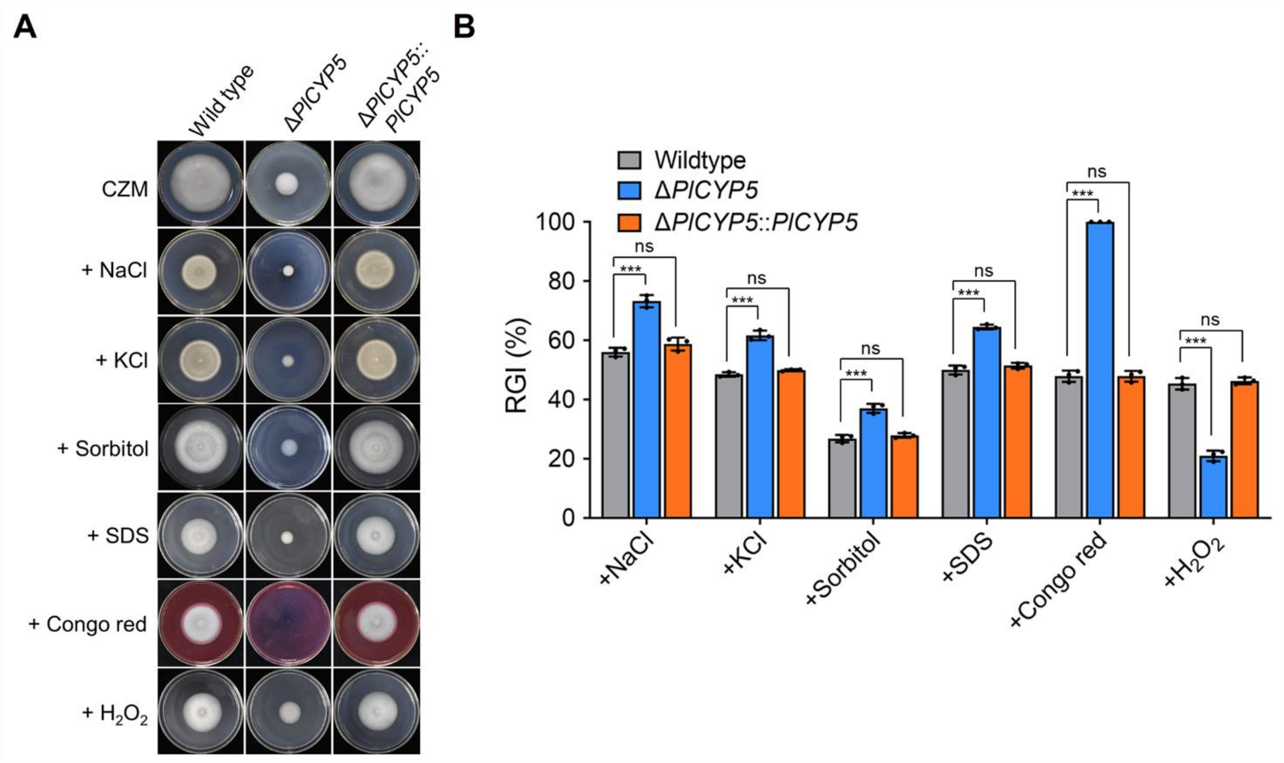

Figure S3. Growth of wildtype, Δ*PlCYP5*, and Δ*PlCYP5*::*PlCYP5* strains to abiotic stresses. **(A)** Colony growth of each strain on normal CZM and CZM supplemented with 1 M NaCl, 1 M KCl, 1.2 M Sorbitol, 0.1% SDS, 0.15 mg/mL Congo red, or 5 mM H_2O_2. Conidia of each strain were inoculated onto the center of CZM plates and incubated at 28 °C for 14 d. **(B)** Relative growth inhibitions of wildtype, Δ*PlCYP5* and Δ*PlCYP5*::*PlCYP5* strains on the CZM containing abiotic stresses versus the normal CZM. The error bars indicate the SD of three replicates. *** denotes $P < 0.001$.

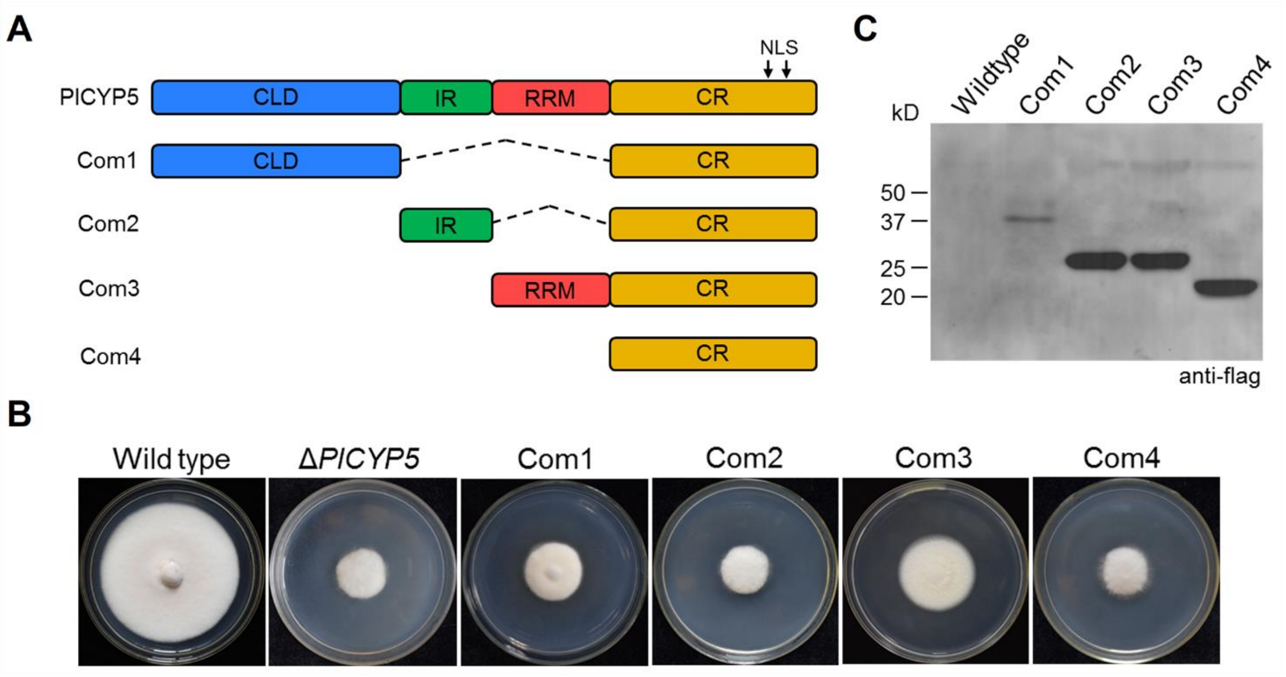

Figure S4. Complementary assay of segmented *PlCYP5* gene in Δ*PlCYP5* strain. **(A)** Schematic diagram of *PlCYP5* gene segmentation design. Each region obtained by domain analysis was fused with the CR containing the nuclear localization signal (NLS). CLD: cyclophilin-like domain, IR: Interval region, RRM: RNA-recognition motif, CR: Charged amino acid region. **(B)** The colony growth of wildtype, Δ*PlCYP5*, and complementary strains on PDA plates cultured for 14 d. **(C)** Expression analysis of the wildtype and complementary strains by western blot using antibody against *flag*.

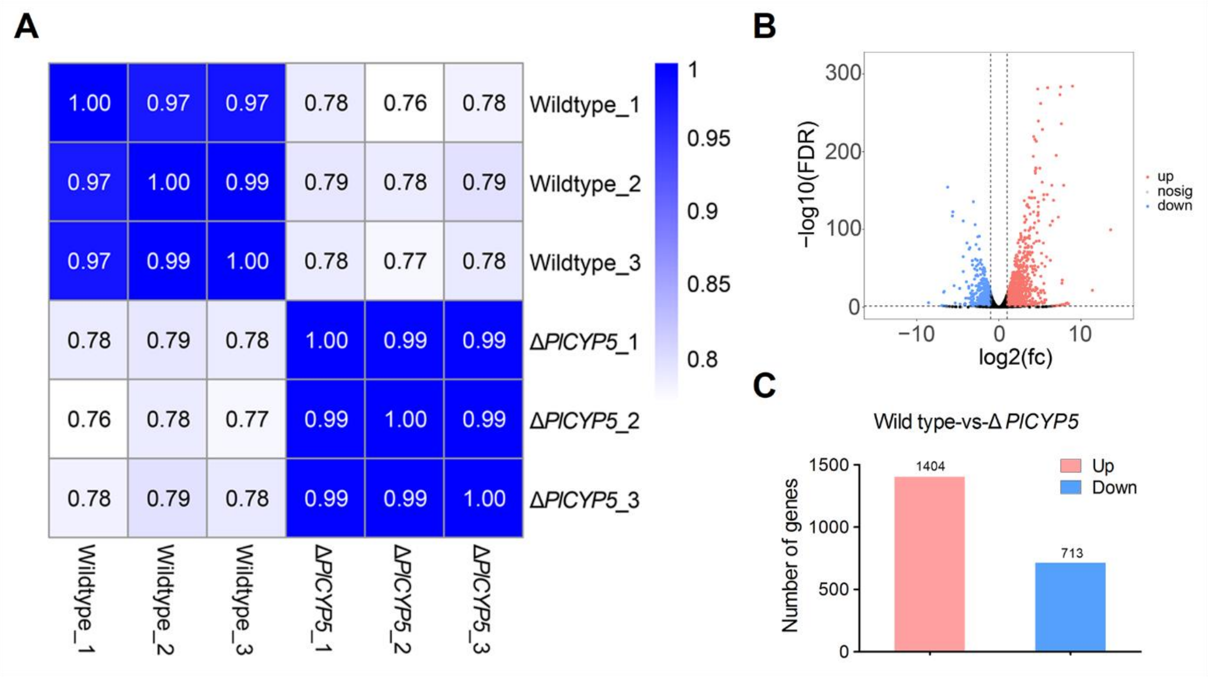

Figure S5. Transcriptome sequencing of wildtype and Δ*PlCYP5*. **(A)** Correlation analysis of wildtype and Δ*PlCYP5* samples based on clean reads. Each strain had three replicates. **(B)** Volcano plot of differential expression genes in Δ*PlCYP5* compared with wildtype. Red and blue dots indicate the up-regulated and down-regulated genes, respectively. **(C)** Numbers of differential expression genes in Δ*PlCYP5* compared with wildtype.

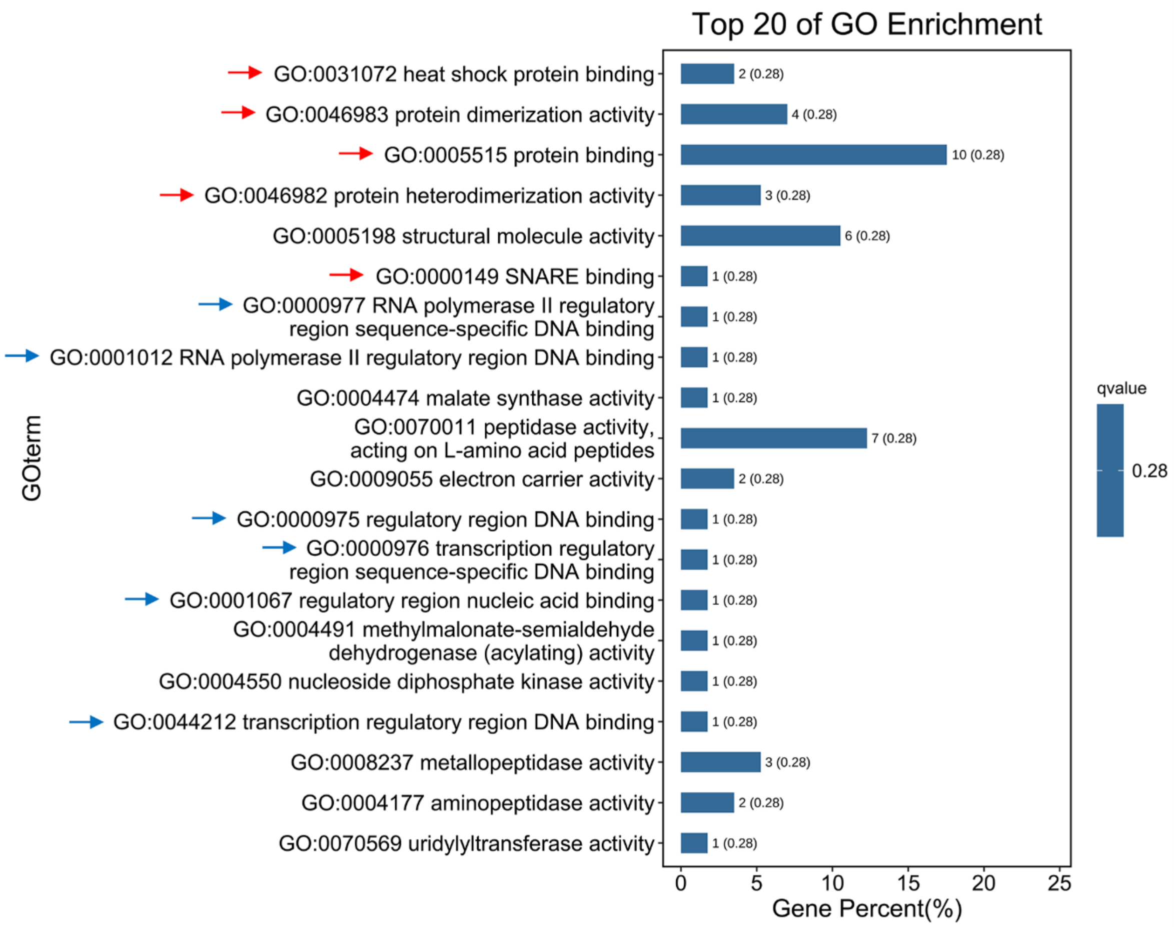

Figure S6. GO enrichment in the molecular function of PlCYP5 interacting candidates. Red and blue arrows indicate the functions related to protein binding and transcriptional regulation, respectively.

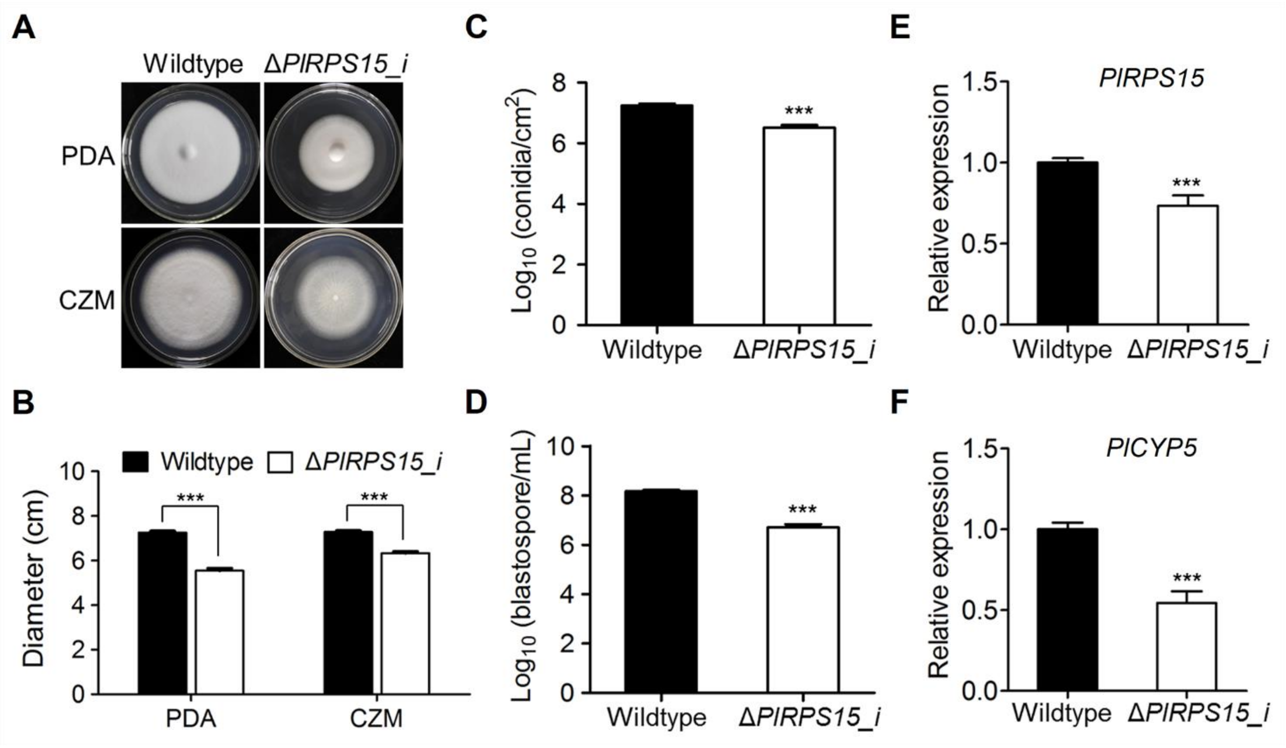

Figure S7. Growth and development phenotypes of wildtype and *PlRPS15* incomplete knockout strain (Δ*PlRPS15_i*). **(A)** Colony growth of each strain on PDA and CZM after 14 d of culture. **(B)** Colony diameters of each strain after 14 d of culture on PDA and CZM. **(C and D)** Conidia **(C)** and blastospore **(D)** yields of each strain. **(E and F)** Relative gene expression of *PlRPS15* **(E)** and *PlCYP5* **(F)** genes in Δ*PlRPS15_i* compared with wildtype. The fold changes of gene expression were calculated by the $2^{-\Delta\Delta Ct}$ method. The error bars indicate the SD of three replicates. *** denotes $P < 0.001$.

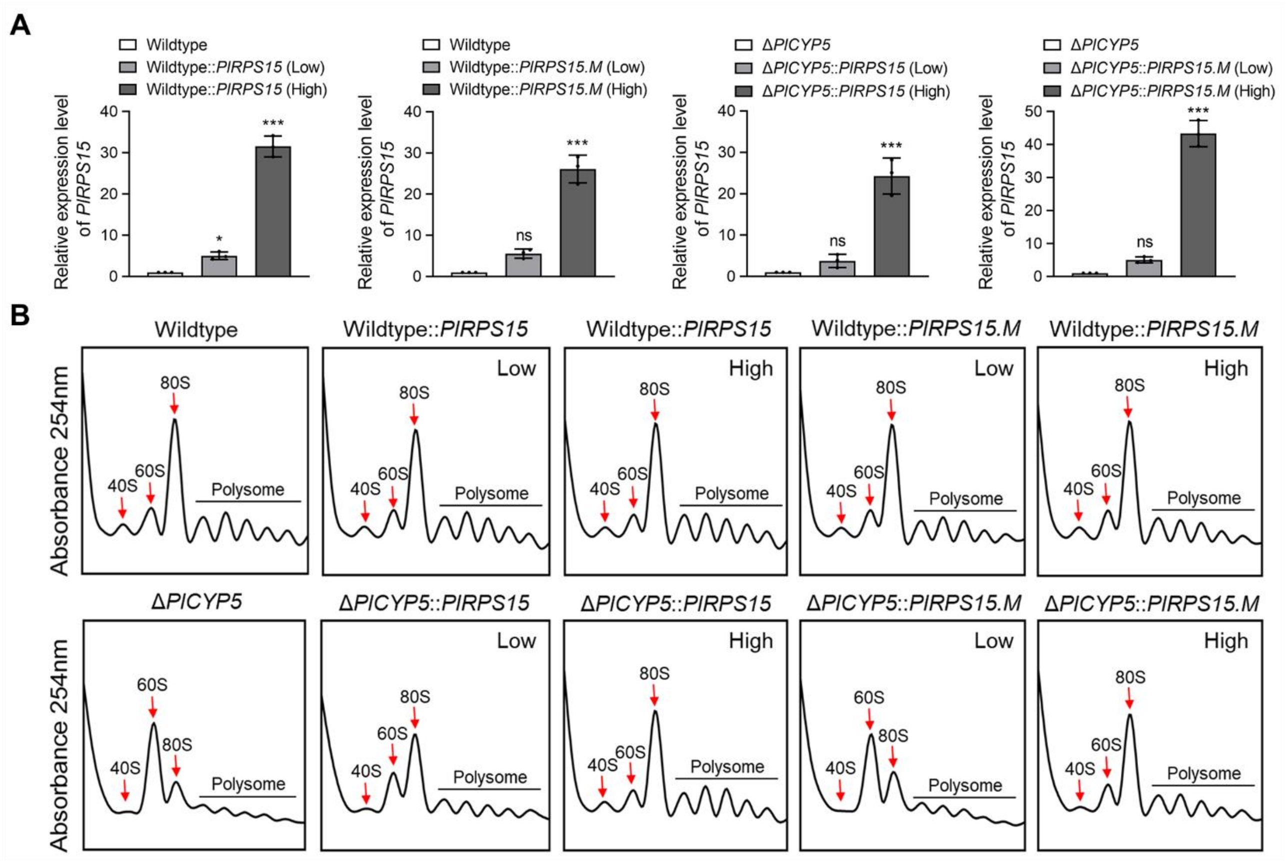

Figure S8. Polysome profiles of wildtype, Δ*PlCYP5*, and strains in low and high expression of the *PlRPS15* or double-point mutated *PlRPS15* (*PlRPS15.M*). **(A)** RT-qPCR verification of the expression level of *PlRPS15* gene in obtained overexpression strains. **(B)** Polysome profiles of wildtype, Δ*PlCYP5*, and overexpression strains. Cell extracts were prepared after cycloheximide treatment and subjected to ultracentrifugal sedimentation on 7-47% sucrose density gradients. Absorbance was recorded at 254 nm. The peaks for 40S, 60S, 80S were indicated by red arrows.

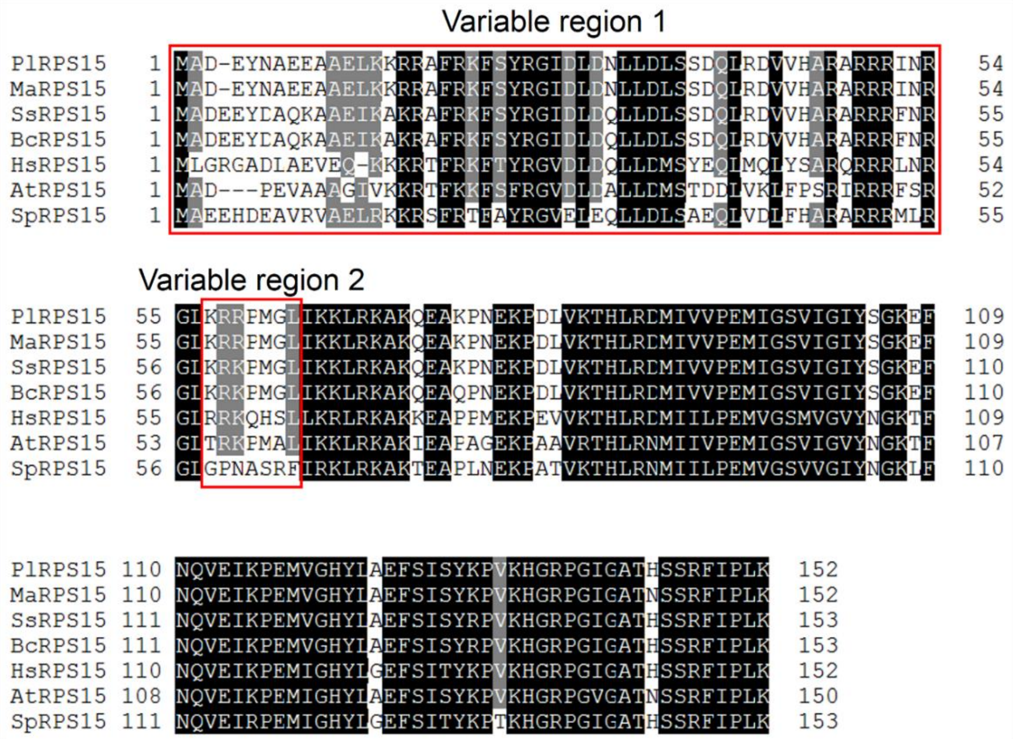

Figure S9. Multiple sequence alignment of RPS15 family proteins between filamentous fungi and other species. Amino acids with different colored background indicate different conservation. The red boxes indicate the two variable regions. Pl: *Purpureocillium lilacinum*, Ma: *Metarhizium anisopliae*, Ss: *Sclerotinia sclerotiorum*, Bc: *Botrytis cinerea*, Hs: *Homo sapiens*, At: *Arabidopsis thaliana*, Sp: *Schizosaccharomyces pombe*.
